# A cell-impermeable kinase inhibitor uncovers outside-in signaling pathways that promote HIV-1 infection

**DOI:** 10.1128/jvi.00160-26

**Published:** 2026-05-11

**Authors:** Kelsey Vinzant, Natalia Cheshenko, Heng Pan, Ronald Cutler, Joshua N. Buckler, Andreas Luxenburger, Lawrence D. Harris, Jeffrey R. Johnson, Steven C. Almo, Betsy C. Herold

**Affiliations:** 1 Department of Microbiology-Immunology, Albert Einstein College of Medicine, Bronx, New York, USA; 2 Department of Pediatrics, Albert Einstein College of Medicine, Bronx, New York, USA; 3 Department of Microbiology, Icahn School of Medicine at Mount Sinai 5925, New York, New York, USA; 4 Global Health and Emerging Pathogens Institute, Icahn School of Medicine at Mount Sinai, New York, New York, USA; 5 Department of Genetics, Albert Einstein College of Medicine, Bronx, New York, USA; 6 Department of Biochemistry, Albert Einstein College of Medicine, Bronx, New York, USA; 7 Ferrier Research Institute, Victoria University of Wellington 428603, Wellington, New Zealand; 8 The Maurice Wilkins Centre for Molecular Biodiscovery, The University of Auckland 1415, Auckland, New Zealand; Icahn School of Medicine at Mount Sinai, New York, New York, USA

**Keywords:** TMEM16F, glypican-1, T cells, kinase inhibitor, HIV-1

## Abstract

Studies of herpes simplex virus (HSV) entry revealed a previously unrecognized “outside-in” signaling pathway involving phosphatidylserine (PS) scrambling and associated translocation and subsequent extracellular activation of canonical intracellular proteins, including Akt. We hypothesized that HIV-1, which activates a different scramblase, TMEM16F, to induce PS externalization, may similarly trigger an “outside-in” signaling response to promote viral entry. To study this process, we utilized a cell-impermeable staurosporine analog, alkyl-CIMSS, which is a broadly active kinase inhibitor that blocks HSV-induced exofacial Akt phosphorylation, and HSV entry. We show that TMEM16F-mediated PS externalization in response to HIV is not associated with Akt translocation; however, surprisingly, pretreatment of cells with alkyl-CIMSS enhanced HIV-1 infection post-entry. To identify potential biological processes that mediated this enhancement, we performed whole-cell total and phosphoproteomics, and bulk RNA sequencing. Cells treated with alkyl-CIMSS exhibited increased cyclin-dependent kinase (CDK) activity, resulting in higher levels of phosphorylated SAMHD1. Further, alkyl-CIMSS treatment robustly upregulated the cell surface density of the proteoglycan glypican-1 (GPC1). Lentivirus-driven GPC1 overexpression or shRNA knockdown demonstrated that, independent of alkyl-CIMSS treatment, GPC1 expression promotes HIV infection. Collectively, these findings demonstrate that alkyl-CIMSS modulates the exofacial plasma membrane to promote susceptibility to HIV infection by increasing CDK activity and upregulating GPC1.

**IMPORTANCE:**

The cell-impermeable pan-kinase inhibitor alkyl-CIMSS inhibits HSV infection by blocking phosphorylation of exofacial Akt. In contrast, alkyl-CIMSS enhances HIV infection. This increase was associated with enhanced cyclin-dependent kinase activity and upregulation of surface-presented glypican-1. These results illustrate that distinct processes associated with the exofacial proteome can either promote viral infection, as observed for HIV, or impede infection, as in the case of HSV, and may facilitate the identification of pathways that can be targeted for future antiviral drug development.

## INTRODUCTION

Viruses usurp host cell machinery to promote their entry and replication (1–3). Prior investigations of herpes simplex virus (HSV) entry into human epithelial cells uncovered a previously unappreciated paradigm associated with activation of phospholipid scramblase 1 (PLSCR1), a calcium (Ca^2+^)-responsive enzyme known to catalyze the movement of phosphatidylserine lipids (PS) between the inner (cytofacial) and outer (exofacial) leaflet of the plasma membrane (PM) (4). Surprisingly, we found that the movement of PS to the exofacial leaflet in response to HSV was associated with the concomitant translocation of intracellular proteins, including the master kinases Akt and phosphoinositide-dependent kinase 1 (PDPK1), as well as other signaling molecules (5, 6). Phosphorylation of exofacial PDPK1 and Akt triggered downstream intracellular signaling responses (“outside-in” signaling), which culminated in fusion of the HSV envelope and the PM, release of viral capsids into the cytoplasm, and their transport to the nuclear pore to initiate viral replication.

To further study this “outside-in” signaling pathway activated by HSV, we designed a cell-impermeable analog of the broadly active kinase inhibitor staurosporine (abbreviated CIMSS) as a tool compound. We confirmed the cell impermeability of CIMSS and demonstrated that it blocked the phosphorylation of exofacial kinases, including Akt, and prevented HSV entry but did not inhibit the phosphorylation of intracellular Akt (6). For example, activation of cytoplasmic Akt in response to insulin binding to the insulin receptor was not perturbed. Vesicular stomatitis virus pseudotyped with SARS-CoV-2 spike protein (VSV-S) and native SARS-CoV-2, but not native VSV, activated a similar exofacial signaling cascade and were also susceptible to CIMSS inhibition.

HIV-1 initiates infection by binding to CD4, with subsequent engagement of either CCR5 or CXCR4 chemokine coreceptors, which trigger Ca^2+^ fluxes (7). The increase in intracellular Ca^2+^ activates a different transmembrane phospholipid scramblase, TMEM16F, a member of the family of Ca^2+^-activated chloride channels and scramblases (CaCCs) (8). Pharmacological inhibition or knockdown of TMEM16F was previously reported to prevent PS translocation and inhibited HIV-1 infection (8). We hypothesized that HIV-mediated activation of TMEM16F might also trigger the exofacial translocation of Akt and render HIV-1 susceptible to inhibition by CIMSS. To test this hypothesis, we compared the susceptibility of HIV-1 and HSV to an analog of CIMSS (alkyl-CIMSS) in cell culture systems and primary peripheral blood mononuclear cells (PBMCs). Consistent with our earlier studies, HSV-induced exofacial movement of both PS and Akt, and infection was inhibited by alkyl-CIMSS. In contrast, HIV-1 triggered exofacial movement of PS but not Akt. Moreover, not only did alkyl-CIMSS fail to inhibit HIV-1 infection, but surprisingly, treatment with alkyl-CIMSS led to an increase in HIV-1 infection, specifically increasing the production of early and late reverse transcriptase products. Phosphoproteomic studies identified increased phosphorylation of multiple cyclin-dependent kinase (CDK) substrates, which was associated with an increase in phosphorylated SAMHD1 in alkyl-CIMSS-treated cells. Transcriptomic and proteomic studies also uncovered a significant increase in glypican-1 (GPC1) in cells treated with alkyl-CIMSS. Lentiviral-driven overexpression or shRNA knockdown of GPC1 showed that GPC1 promotes HIV-1 infection independent of alkyl-CIMSS treatment. Flavopiridol, a CDK inhibitor, overcame the effects of both alkyl-CIMSS and GPC1 overexpression on HIV infection.

## RESULTS

### HIV-1 triggers exofacial translocation of phosphatidylserine lipids but not Akt

**Fig 1:**
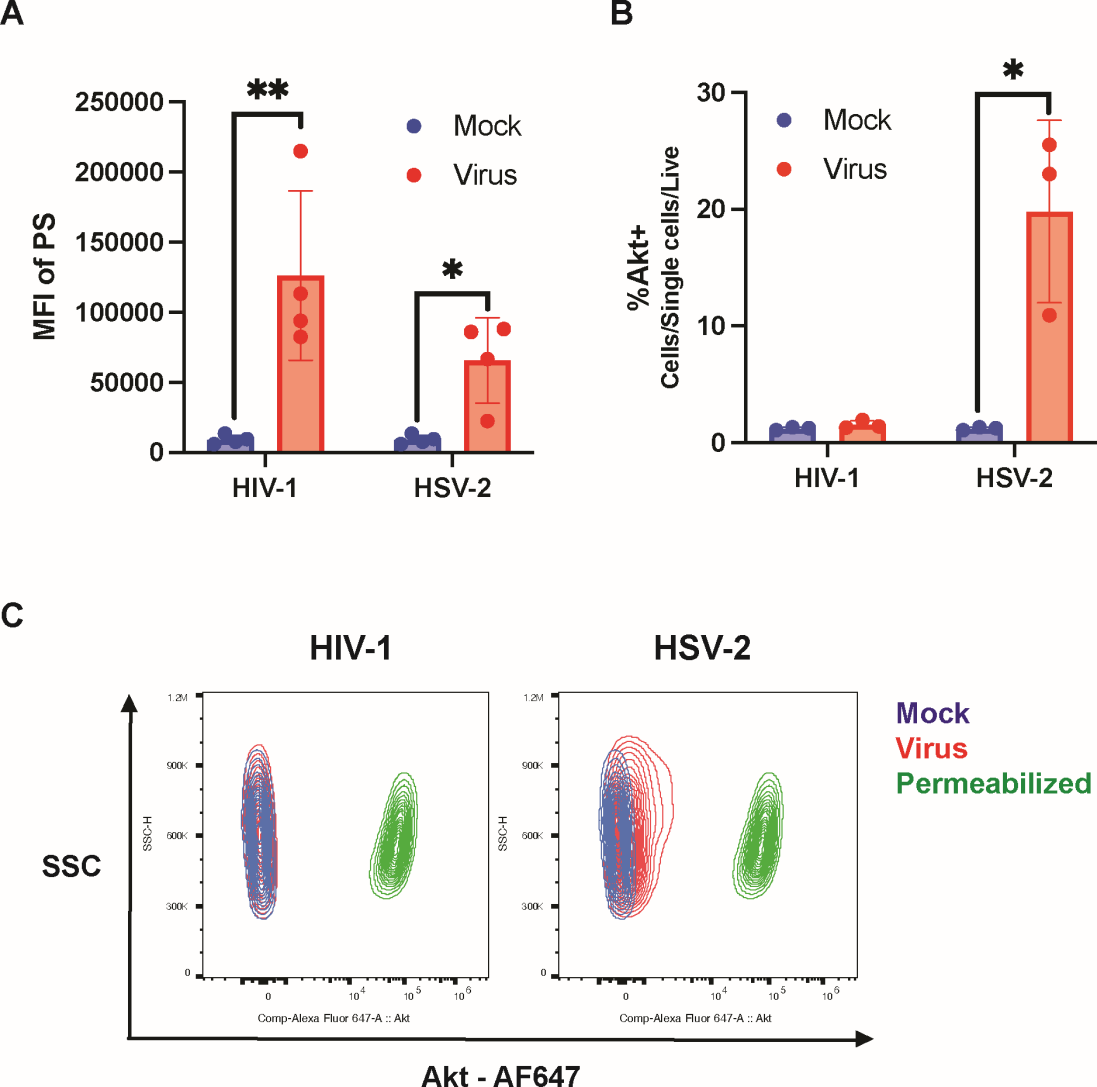
HIV and HSV trigger exofacial translocation of phosphatidylserines, but only HSV increases exofacial Akt. (**A**) TZM-bl cells were treated with HIV-1 or HSV-2 for 30 min and stained for cell viability and phosphatidylserines (PS). Results are presented as the mean fluorescence intensity (MFI) of PS after gating on the live cell population (*n* = 4 independent experiments). (**B**) Cells were infected as in panel A, and the percentage of non-permeabilized live cells expressing Akt was quantified by flow (*n* = 3 independent experiments). (**C**) Representative contour plots showing changes in Akt staining following virus treatment (blue: mock, red: virus treated, green: permeabilized). Results are shown as mean ± SD and compared by unpaired *t*-test; *P < 0.05, **P < 0.01.

We previously found that HSV and SARS-CoV-2 activate PLSCR1 to trigger the exofacial translocation of PS and Akt with subsequent phosphorylation of extracellular Akt to promote viral entry; inhibition of Akt phosphorylation by the cell-impermeable pan-kinase inhibitor, CIMSS, reduced viral entry (5, 6, 9). HIV-1 activates a different phospholipid scramblase, TMEM16F, to induce PS externalization and promote entry (8). We hypothesized that activation of TMEM16F might also trigger translocation of Akt and its subsequent phosphorylation, rendering HIV-1 susceptible to CIMSS inhibition. To directly test this notion, we treated TZM-bl cells, a HeLa cell line engineered to express CD4, CCR5, CXCR4, and a luciferase reporter under the control of the HIV-1 long terminal repeat (LTR), with HSV-2 or HIV-1 and assayed for exofacial PS and Akt. Both HSV-2 and HIV-1 triggered a significant increase in exofacial PS within 30 min of exposure (Fig. 1A), but only HSV-2 exposure resulted in detectable exofacial Akt as assayed by flow cytometry of non-permeabilized cells (Fig. 1B and C). Permeabilized cells were included as a positive control for detection of intracellular Akt.

**Fig 2:**
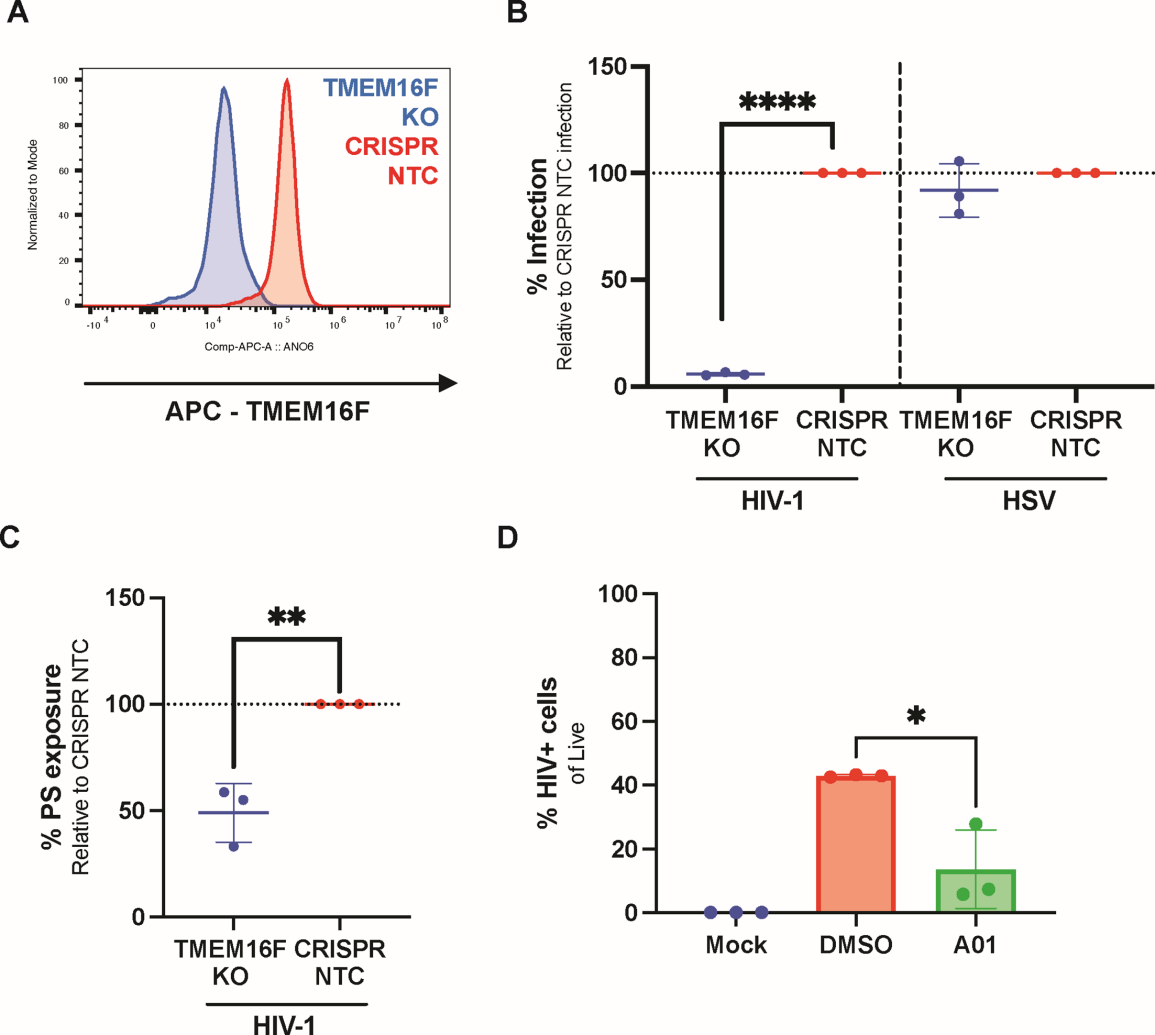
HIV but not HSV infection is reduced in the absence of TMEM16F. (**A**) Representative histogram showing TMEM16F expression in TMEM16F knockout (KO) (blue) and CRISPR non-targeting control (NTC) cells (red). (**B**) TMEM16F KO or control cells were infected with HIV-1 or HSV-VP26-mCherry and infection quantified by assessing the percentage of p24 or mCherry positive cells by flow cytometry 72 or 4 h post-infection, respectively. Results are presented as the percent of HIV or HSV+ cells relative to control cells (*n* = 3 independent experiments each conducted in duplicate). (**C**) TMEM16F KO or control cells were exposed to HIV-1_BaL_ for 30 min and then stained for exofacial PS by flow cytometry in non-permeabilized cells. Results are presented as the MFI of PS on HIV-exposed cells normalized to the virus exposed control cells (*n* = 3 independent experiments each conducted in duplicate). (**D**) Jurkat-CCR5 cells were treated with 60 μM CaCCinh-A01 (A01) or DMSO vehicle control before infection with 5 ng p24 HIV-1_BaL_. Cells were fixed and permeabilized for p24 and viability staining after 72 h of incubation (*n* = 3 independent experiments each conducted in duplicate). Results are presented as percent of live cells. Results are shown as mean ± SD and compared by unpaired *t*-test; *P < 0.05, **P < 0.01, ****P <0.0001.

We hypothesized that the difference in Akt translocation may be explained, in part, by the findings that HSV activates PLSCR1 (5), whereas HIV-1 has been previously shown to activate TMEM16F (8). To confirm that HIV-1 utilizes TMEM16F, we engineered a Jurkat-CCR5 cell line in which TMEM16F was knocked out (KO) using CRISPR technology. The KO was confirmed by assessing gene and protein expression by RT-qPCR and flow cytometry, respectively (Fig. 2A). Compared to control cells, exposure of the TMEM16F KO cells to HIV-1 (5 ng p24) resulted in a 94 ± 0.44% reduction in HIV-1 infection as measured by the percentage of cells expressing viral p24 at 72 h (*P* < 0.0001). In contrast, the KO did not block HSV entry, which was quantified 4 h post-exposure using a reporter virus expressing mCherry linked to the HSV capsid protein VP26 (Fig. 2B). In addition, significantly less exofacial PS was detected by flow cytometry following HIV-1 exposure of the TMEM16F KO compared to the CRISPR non-targeting control cells (CRISPR NTC) (Fig. 2C). To further validate a role for TMEM16F in HIV-1 infection, we examined the effects of the pharmacological inhibitor CaCCinh-A01 (A01), which blocks TMEM16F scramblase activity, on viral infection (10, 11). Treatment of Jurkat-CCR5 cells with A01 (Fig. 2D) resulted in a significant reduction in HIV-1 infection as measured by the percentage of cells expressing p24 at 72 h.

### A cell-impermeable analog of staurosporine enhances HIV-1 infection

We previously described acyl-CIMSS, a cell-impermeable staurosporine analog in which an acyl sulfonate is installed at the 4′-secondary amine via an amide linkage. As the amide linkage may reduce binding affinity due to unfavorable steric interactions and loss of hydrogen bonding potential (12), we generated an alkyl CMISS analog via 4′-amine-alkylation, which would be less subject to these liabilities (Fig. S1 and S2). Additionally, an alkyl linkage would be expected to provide greater stability than an amide bond. These analogs exhibited nearly indistinguishable cell impermeability and kinase inhibition properties (Table S1). Consistent with its cell impermeability, alkyl-CIMSS had no discernible effects on cell growth or viability when TZM-bl cells, Jurkat-CCR5, or primary human CD4+ T cells were incubated with the drug for 24 h (Fig. S1B). Moreover, unlike unmodified staurosporine, alkyl-CIMSS did not induce apoptosis as assessed by caspase activation (Fig. S1C).

**Fig 3:**
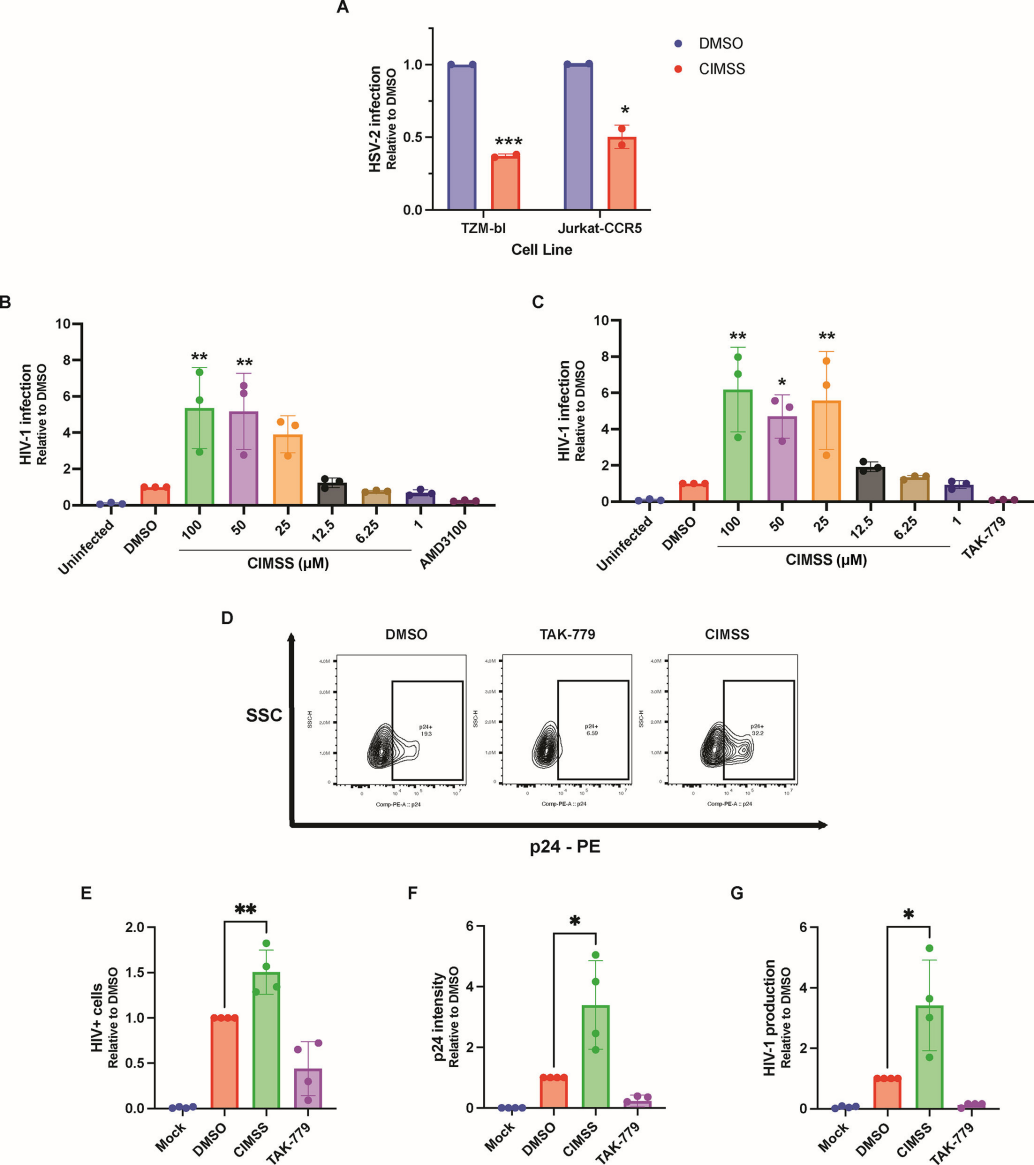
Alkyl-CIMSS inhibits HSV-2 but enhances HIV-1 infection. (**A**) The indicated cells were treated with 10 μM alkyl-CIMSS or DMSO vehicle control and then infected with HSV-2(G) (10 pfu/cell) for 4 h. Infection was monitored quantifying ICP0 expression in isolated RNA (*n* = 2 independent experiments each conducted in duplicate per cell type). (**B and C**) TZM-bl cells were treated with the indicated concentrations of alkyl-CIMSS, DMSO vehicle control, 10 μM TAK-779 (R5), or 10 μM AMD3100 (X4) and then infected with 1.5 ng p24 of HIV-1_BaL_ (R5) (**B**) or HIV-1_iiiB_ (X4) (**C**). Infection was quantified by lysing cells for luciferase expression after 48 h incubation (*n* = 3 independent experiments each conducted in duplicate for each viral strain). (**D–F**) Jurkat-CCR5 cells were treated with 50 μM CIMSS, DMSO vehicle control, or 10 μM TAK-779 and infected with 5 ng p24 HIV-1_BaL_. At 72 hpi, cells were fixed and permeabilized for p24 and stained for cell viability. A representative flow plot is shown in panel D, percentage p24+ cells in panel E, and mean fluorescence intensity in panel F (*n* = 4 independent experiments each conducted in duplicate). (**G**) Viral yields from infected Jurkat-CCR5 cells from panel D were quantified by TZM-bl luciferase assay. Results are presented as mean ± SD relative to cells infected in the presence of the DMSO vehicle control and compared by one-way ANOVA (B and C) or unpaired *t*-test (A, E–G); *P < 0.05, **P < 0.01, ***P < 0.001.

At a dose of 10 μM (which is the approximate dose of acyl-CIMSS that inhibited HSV infection by 50%), alkyl-CIMSS reduced HSV-2 infection of TZM-bl and Jurkat-CCR5 cells by a mean of 63 ± 0.01% and 50 ± 0.06% (*n* = 2), respectively, relative to vehicle (DMSO)-treated cells. Infection was assessed by quantifying intracellular ICP0 expression, an immediate early HSV gene, 4 h post-viral exposure (Fig. 3A). Given that HIV-1 did not induce Akt translocation during viral-induced host plasma membrane reorganization, we hypothesized that HIV-1 would not be susceptible to CIMSS inhibition. Surprisingly, not only did alkyl-CIMSS fail to inhibit HIV-1 infection, but the compound significantly increased both HIV-1_IIIB_ (CXCR4-utilizing strain) and HIV-1_BaL_ (R5-utilizing strain) infection of TZM-bl cells in a dose-dependent manner relative to cells treated with DMSO control (Fig. 3B and C). AMD3100, a CXCR4 antagonist, and TAK-779, a CCR5 antagonist, were included as negative controls. Based on the dose response in TZM-bl cells, for subsequent experiments, we selected 50 µM alkyl-CIMSS, which enhanced HIV-1_BaL_ infection 4.8 ± 2.3-fold, and focused on CCR5-utilizing viruses, which are responsible for most primary HIV-1 infections (13). Consistent with the TZM-bl results, alkyl-CIMSS significantly increased HIV-1_BaL_ infection of Jurkat-CCR5 cells, which was quantified both by directly measuring p24 expression by flow cytometry or indirectly, by collecting culture supernatants and quantifying viral yields in a TZM-bl luciferase assay (Fig. 3D through G).

### Alkyl-CIMSS acts on the cell to promote expression of reverse transcriptase products

**Fig 4:**
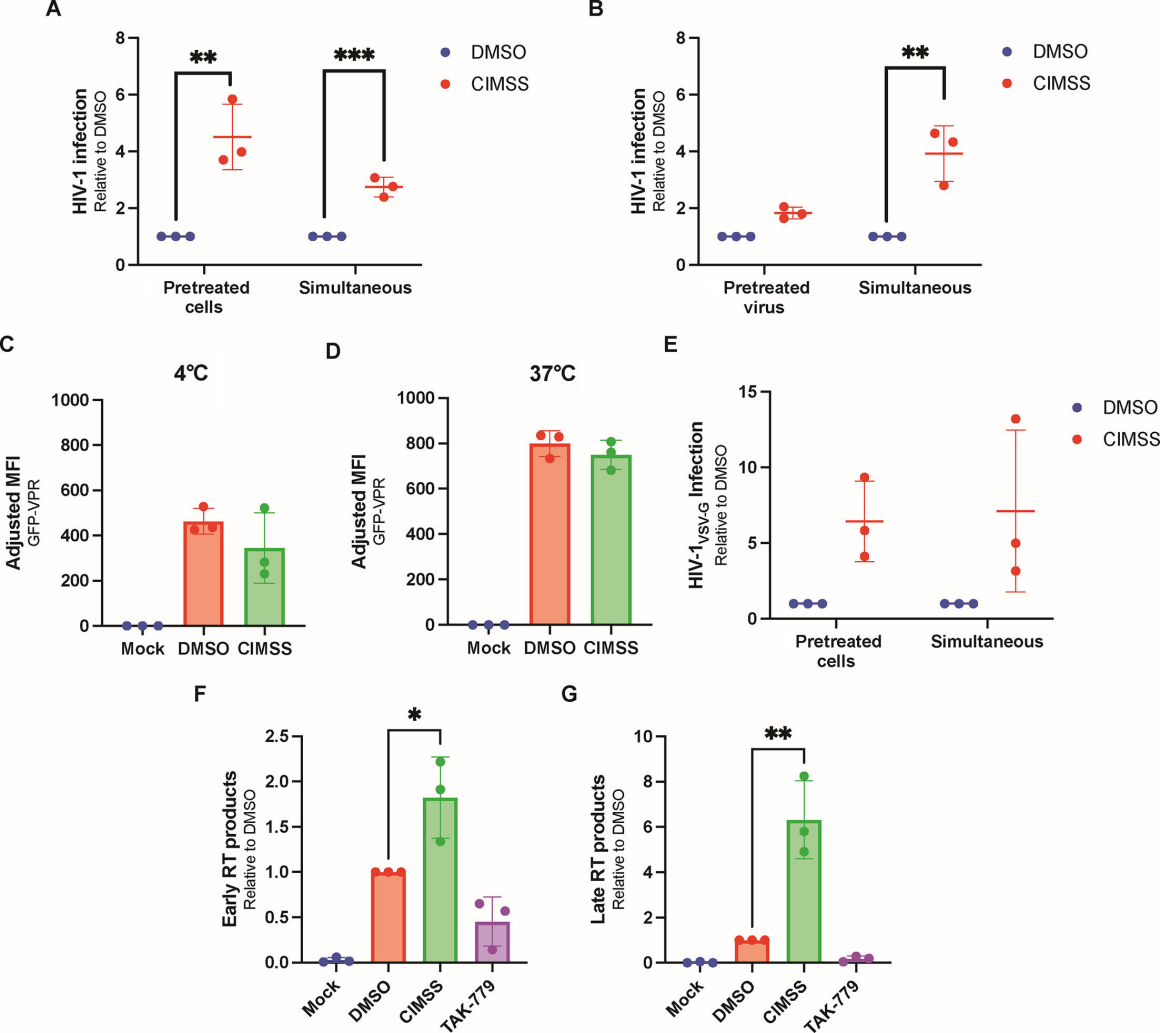
Alkyl-CIMSS acts on cells prior to viral exposure to promote the production of early and late gene transcripts. (**A**) TZM-bl cells were pretreated with alkyl-CIMSS or DMSO vehicle control for 1 h and then washed before infection with 1.5 ng p24 HIV-1_BaL_, or (**B**) concentrated HIV-1_BaL_ was treated with for 1 h and then the virus-drug mixture was diluted 40-fold (final alkyl-CIMSS concentration 1 μM) prior to infecting TZM-bl cells. In parallel, cells were exposed simultaneously to virus and compound. Cells were harvested 72 hpi and analyzed for luciferase activity. Results are presented as fold change over infection in the presence of DMSO (*n* = 3 independent experiments each conducted in triplicate). (**C and D**) Jurkat-CCR5 cells were treated with 50 μM alkyl-CIMSS or vehicle control and exposed to HIV-1 carrying a GFP-VPR fusion protein for 2 h either at 4℃ or 37℃ prior to being washed extensively. Cell-associated virus was quantified by measuring GFP expression by flow cytometry, and results are presented as the MFI GFP after subtracting background from mock-infected cells (*n* = 3 independent experiments each conducted in duplicate). (**E**) TZM-bl cells were pretreated with 50 μM alkyl-CIMSS or vehicle control before or infected simultaneously with HIV-1_VSV-G_ (1.5 ng p24). Infection was monitored by assaying for luciferase activity 72 h (*n* = 3 independent experiments each conducted in triplicate). (**F and G**) Jurkat-CCR5 cells were treated with 50 μM alkyl-CIMSS, 10 μM TAK-779, or DMSO control before infection with 5 ng p24 HIV-1_BaL_. At 6 and 24 hpi, cells were harvested for DNA and quantification of early and late reverse transcription products, respectively (*n* = 3 independent experiments each conducted in duplicate). In panels E–G, results are presented as mean ± SD fold change over infection in the presence of DMSO and compared to the DMSO control by unpaired *t*-test; *P < 0.05, **P < 0.01, ***P < 0.001.

To assess the impact of alkyl-CIMSS on HIV-1 infection, we tested whether the compound targets a molecule accessible on the cell surface prior to, or only following HIV-1 exposure, or whether it directly interacts with viral particles. TZM-bl cells were pretreated with 50 μM alkyl-CIMSS (or control buffer) for 1 h and then washed extensively prior to HIV-1 infection (1.5 ng p24), or concentrated virus (60 ng p24) was pretreated with 50 μM alkyl-CIMSS (or control buffer) for 1 h and then diluted 40-fold to 1.5 ng p24 and an inactive final concentration of 1.25 μM alkyl-CIMSS before infecting cells. As a comparator in both assays, the cells were exposed simultaneously to virus and alkyl-CIMSS. Enhancement of HIV-1 infection persisted and was slightly greater (*P* = 0.06) if cells were pretreated with alkyl-CIMSS and then washed prior to infection (Fig. 4A) but was reduced when virus was pretreated with the drug and diluted (Fig. 4B), suggesting that the compound affects a cell surface molecule that is accessible prior to viral exposure or TMEM16F activation.

To determine if alkyl-CIMSS promotes HIV-1 binding, entry, or acts post-entry, we took advantage of a virus carrying a Vpr-enhanced green fluorescence protein fusion protein (Vpr-EGFP), which is packaged into the virion during assembly, allowing for detection of bound and internalized virus. Jurkat-CCR5 cells were pretreated with 50 μM alkyl-CIMSS or control buffer for 4 h and then exposed to the Vpr-EGFP viral construct for 2 h at 4°C, a temperature only permissive for viral binding. The cells were then washed, and bound virus was detected by assaying for cell-bound EGFP. Alternatively, after the 2-hour viral binding period, the cells were shifted to 37°C for an additional 2 h to allow viral entry. No differences in EGFP were detected in the absence or presence of alkyl-CIMSS (Fig. 4C and D), indicating that the CIMSS-induced enhancement is post-entry. To further examine this behavior, we assessed the effects of alkyl-CIMSS on a single-cycle VSV-G-pseudotyped HIV-1 virus, which binds to cells primarily through interactions with the low-density lipoprotein receptor and enters by endocytosis. Alkyl-CIMSS increased VSV-G-pseudotyped HIV infection as measured by luciferase production (6.4 ± 2.7-fold), both when TZM-bl cells were pretreated with the compound or when the VSV-G-pseudotyped virus and the compound were added simultaneously (Fig. 4E).

Following entry, HIV-1 employs reverse transcriptase to produce reverse transcription intermediates that support viral integration. To assess whether CIMSS impacts these processes, Jurkat-CCR5 cells were again infected with HIV-1_BaL_ in the presence of alkyl-CIMSS or control DMSO buffer, and DNA was isolated for quantification of early and late reverse transcription products. TAK-779 was included as an inhibitory control. Alkyl-CIMSS significantly increased the amount of early reverse transcription products 6 h post-infection and late reverse transcription products 24 h post-infection, whereas cells infected in the presence of TAK-779 had lower levels of both early and late reverse transcription products, reflecting less virus entering the cell (Fig. 4F and G).

### Alkyl-CIMSS induces an increase in cyclin-dependent kinase (CDK) activity

**Fig 5:**
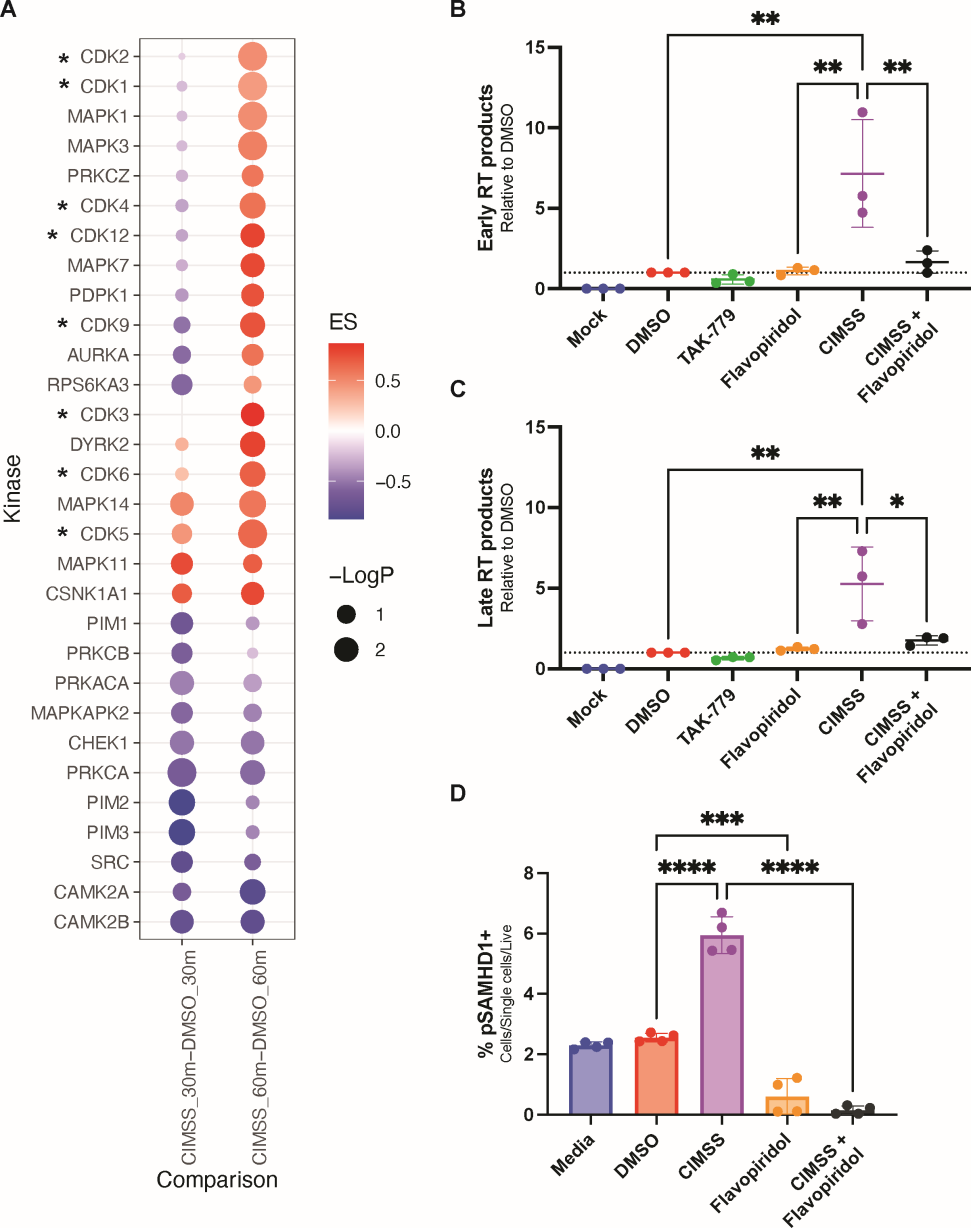
Alkyl-CIMSS treatment promotes CDK activity and phosphorylation of SAMHD1. (**A**) Kinase substrate enrichment analysis (KSEA) of substrates identified following phosphoproteomic analysis of Jurkat-CCR5 cells treated with DMSO or alkyl-CIMSS for 30 and 60 min. Kinases shown have a *P*-value < 0.05. (**B and C**) Jurkat-CCR5 cells were treated with DMSO, 10 μM TAK-779, 10 μM flavopiridol, 50 μM alkyl-CIMSS, or a combination of flavopiridol and alkyl-CIMSS before infection with 5 ng p24 HIV-1_BaL_. At 24 hpi, cells were harvested for DNA and quantification of early (**B**) and late reverse transcription (**C**) products. Results presented as mean ± SD fold change over infection in the presence of DMSO (*n* = 3 independent experiments conducted in duplicate). (**D**) Jurkat-CCR5 cells were treated with DMSO, 50 μM alkyl-CIMSS, 10 μM flavopiridol, or a combination of flavopiridol and alkyl-CIMSS overnight and stained for intracellular pSAMHD1 (Thr592). Representative flow plot shown (*n* = 4 independent experiments conducted in duplicate). Results from panel D presented as percent of pSAMHD1+ live cells. Results in panels B and C are compared by one-way ANOVA; *P < 0.05, **P < 0.01, ***P < 0.001, ****P < 0.000 1.

Since alkyl-CIMSS is a cell-impermeable kinase inhibitor, we hypothesized that it might trigger an “outside-in” signaling response and modulate phosphorylated proteins within the cell to promote HIV reverse transcription. We performed whole-cell phosphoproteomics comparing Jurkat-CCR5 cells treated with alkyl-CIMSS or DMSO control in the absence of viral infection (Table S2). Kinase substrate enrichment analysis (KSEA) revealed an inferred decrease in the activity of several kinases at 30 min, but more strikingly, at 60 min, multiple CDKs had higher levels of inferred activity relative to the DMSO-treated control cells (Fig. 5A). To test if this CDK activity was associated with enhanced HIV infection, we added flavopiridol, a pan-CDK inhibitor, to the alkyl-CIMSS or control virally exposed cells. Flavopiridol overcame the enhancing effects of alkyl-CIMSS on early and late reverse transcription products but had no significant effect on the control-infected cells (Fig. 5B and C).

CDKs 1, 2, 4, and 6 have been linked to HIV-1 reverse transcription activity through their ability to phosphorylate the host restriction factor SAMHD1 (14, 15). To assess this potential contribution, we measured pSAMHD1 in alkyl-CIMSS-treated versus DMSO Jurkat-CCR5 cells after an overnight incubation. Significantly more pSAMHD1-positive cells were detected by flow cytometry in alkyl-CIMSS-treated cells compared to DMSO-treated cells (5.9 ± 0.61% vs 2.6 ± 0.14%, *P* < 0.0001), and the response was blocked by the addition of flavopiridol (Fig. 5D and E).

### Alkyl-CIMSS enhancement of HIV-1 infection is linked to upregulation of GPC1

**Fig 6:**
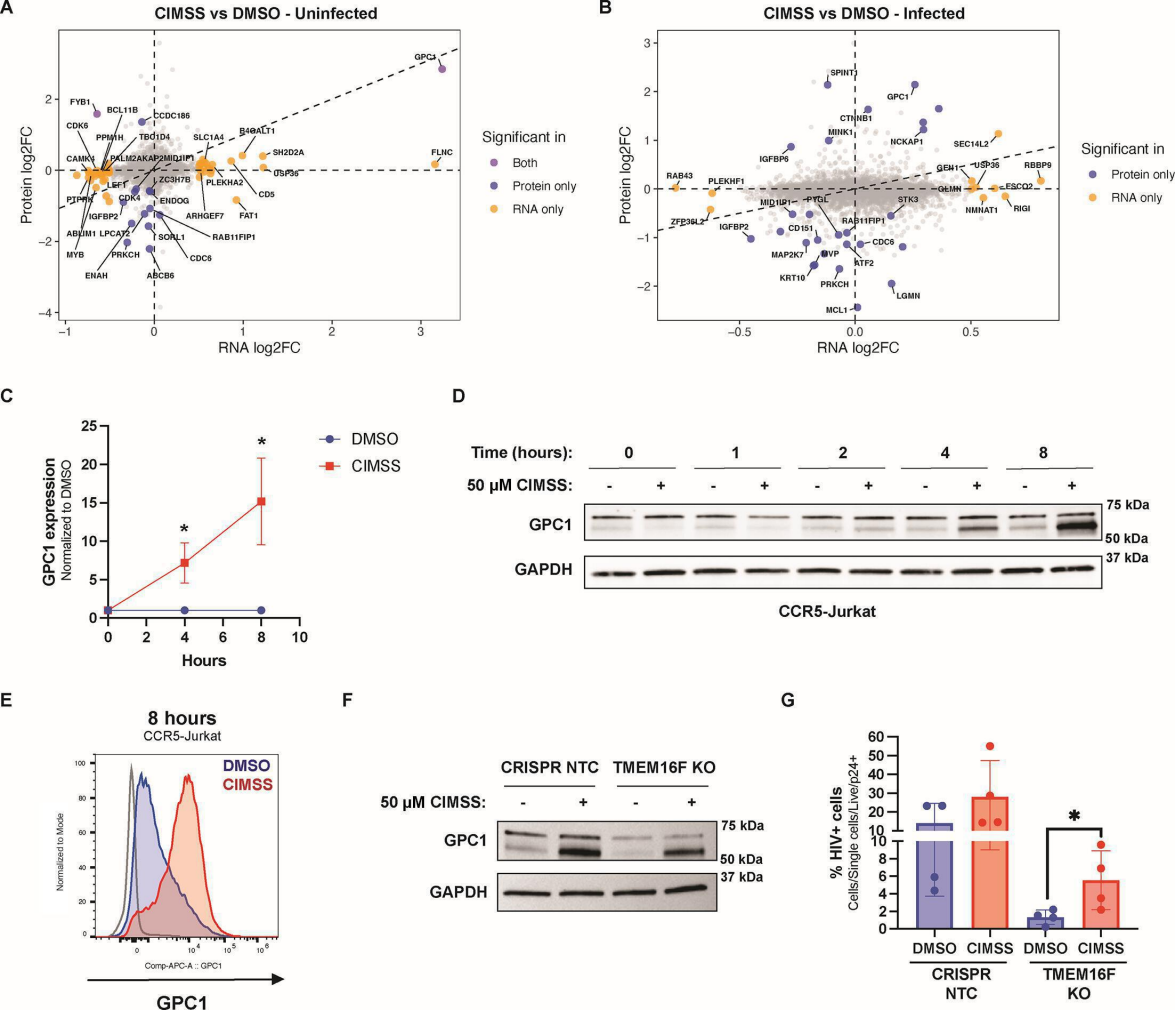
Transcriptomics and proteomics identify increased glypican-1 expression in response to alkyl-CIMSS treatment. (**A**) Jurkat-CCR5 cells were treated with 50 μM alkyl-CIMSS or DMSO buffer or (**B**) subsequently infected with HIV. The cells were lysed after 8 h and processed for proteomics and RNA sequencing. The X-axis indicates the log2 fold-change (FC) in RNA, and the Y-axis indicates log2FC in protein expression. A transcript or protein was considered significant if the adjusted *P*-value was < 0.05 or log2FC > 0.5. Purple denotes significant changes at both RNA and protein levels, blue denotes significance only at the protein level, and yellow denotes significance only at the RNA level. (**C**) Jurkat-CCR5 cells were treated with 50 μM alkyl-CIMSS or vehicle control for the indicated times, and GPC1 gene expression or protein expression monitored by RT-qPCR (*n* = 3) or (**D**) immunoblotting (*n* = 2). (**E**) Jurkat-CCR5 (*n* = 2 independent experiments) were treated as in panel C, and GPC1 was quantified at 8 h by flow cytometry in non-permeabilized cells. Histogram depicts the MFI of exofacial GPC1 on DMSO (blue) or alkyl-CIMSS (red)-treated cells 8 h post-treatment. (**F**) TMEM16F KO or CRISPR control cells were treated with alkyl-CIMSS or DMSO control for 8 h. Cells were harvested and analyzed for GPC1 protein expression by western blotting or (**G**) infected with HIV-1_BaL_ and stained for intracellular p24 72 hpi. Results are presented as percent of live, p24+ cells (*n* = 4). Results in panels C and G are presented as mean ± SD and compared by unpaired *t*-test; *P < 0.05,

To further explore how alkyl-CIMSS affects the cells to promote HIV infection, we also performed bulk RNA sequencing and whole-cell proteomics. Jurkat-CCR5 cells were exposed to alkyl-CIMSS or DMSO in the absence or presence of HIV-1_BaL_ and lysed after 8 h, a time point corresponding to the increase in reverse transcription products. Differentially expressed gene transcripts and proteins were defined by log2 fold-change (FC) > 0.5 and adjusted *P*-value < 0.05. Applying these criteria, glypican 1 (GPC1) was identified as the most significantly upregulated RNA (FC = 3.2, adj. *P*-value = 1.0E-211) and protein (FC = 2.8, adj. *P*-value = 1.5E-5) in alkyl-CIMSS-treated vs DMSO-treated HIV-1-uninfected cells (Fig. 6A; Table S3). GPC1 was also among the most upregulated proteins identified in the HIV-1-infected cells (FC = 2.1, adj. *P*-value = 0.0015, Fig. 6B; Table S4). Notably, neither CD4 nor CCR5 were increased in response to alkyl-CIMSS (Table S2).

We confirmed that alkyl-CIMSS increased GPC1 by directly quantifying gene and protein expression. Within 4 h of exposure, alkyl-CIMSS treatment significantly increased *GPC1* mRNA levels relative to control-treated cells (7.2 ± 2.6-fold, *P* < 0.05) (Fig. 6C). Immunoblots revealed two GPC1 protein species, with a significant time-dependent increase in the lower (~60 kDa) band following alkyl-CIMSS treatment, which presumably reflects newly synthesized protein lacking complete post-translational glycosylation (Fig. 6D). There was also an increase in cell surface GPC1 detected by flow cytometry on non-permeabilized cells following alkyl-CIMSS treatment, consistent with GPC1 being a membrane protein (Fig. 6E). The addition of alkyl-CIMSS also increased GPC1 expression in TMEM16F KO cells, which was associated with a significant increase in HIV-1 infection (*P* <0.05) (Fig. 6F and G). However, infection remained lower in the KO compared to the CRISPR NTC cells after alkyl-CIMSS treatment, consistent with the role of TMEM16F in HIV entry.

### GPC1 enhances HIV-1 transcription

**Fig 7:**
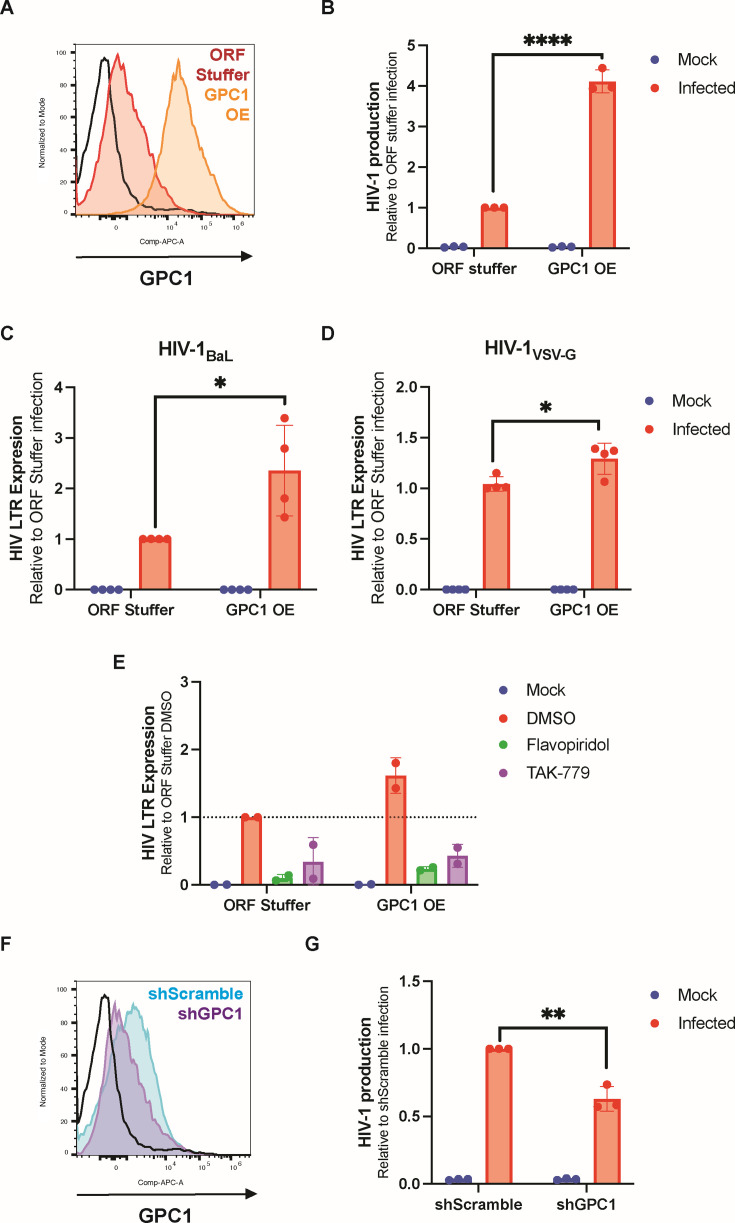
Changes in glypican-1 expression modulate HIV-1 infection post-entry. (**A**) Jurkat-CCR5 cells were transduced with a full-length glypican-1 overexpression (GPC1 OE) or control (ORF stuffer) construct. GPC1 overexpression was assessed by flow cytometry. (**B**) OE or control cells were infected with HIV-1_BaL_, and supernatant was collected 72 hpi for measurement of HIV-1 production by TZM-bl assay (*n* = 3). (**C**) OE or control cells were infected with HIV-1_BaL_ or (**D**) HIV-1_VSV-G_ (2 ng p24) and harvested at 48 and 24 h, respectively, for measurement of LTR expression by RT-qPCR (*n* = 4). (**E**) As in panel C, OE or control cells were infected with HIV-1_BaL_ in the presence of either DMSO, 10 μM flavopiridol, or 10 μM TAK-779 and harvested at 48 hpi for measurement of LTR expression (*n* = 2). (**F**) Jurkat-CCR5 cells were transduced with a GPC1 shRNA (shGPC1) or scramble shRNA (shScramble) construct; silencing was assessed by flow cytometry. (**G**) shGPC1 or shScramble cells were infected with HIV-1_BaL_, and supernatant was collected 72 hpi for measurement of HIV-1 production by TZM-bl assay (*n* = 3). Results are presented as fold change over infection of control cells in panels B–E and G. All results are presented as mean ± SD and compared by unpaired *t*-test; *P < 0.05, **P < 0.01, ****P < 0.0001.

To test whether the increase in GPC1 protein directly contributes to increased HIV-1 infection, Jurkat-CCR5 cells were transduced with a full-length GPC1 or control lentiviral construct expressing mCherry. The cells were then sorted, and overexpression of GPC1 at the cell surface was confirmed by flow cytometry. Cells transduced with the GPC1 overexpression construct showed a 32-fold increase in GPC1 median fluorescence intensity (MFI) relative to control cells (Fig. 7A). The transduced cells were infected with HIV-1 (5 ng p24), and after 72 h, culture supernatants were harvested and viral yields quantified by TZM-bl assay. There was a significant increase in HIV-1 production in GPC1-transduced cells compared to control cells (4.1 ± 0.3-fold, P < 0.001) (Fig. 7B). Because GPC1 was introduced using a lentivirus, we could not use early or late gene transcripts to assess this step in HIV infection. Instead, we used HIV LTR expression as an early marker of viral infection after validating that the GPC1 lentiviral vector does not express LTR RNA following integration. GPC1 overexpression significantly increased HIV LTR expression when cells were infected with either HIV-1_BaL_ or HIV-1_VSV-G_ (*P* < 0.05), suggesting that the effects of GPC1 overexpression are independent of viral entry since the VSV-pseudotyped virus enters by a different pathway. The increased expression of HIV LTR was lost when flavopiridol was added to the GPC1-overexpressing cells (Fig. 7E). Flavopiridol also reduced HIV transcription in the control cells, consistent with its inhibitory effects on viral transcription (16).

The role of GPC1 in HIV-1 infection was further tested using a knockdown model. Jurkat-CCR5 cells were transduced with an mCherry+ GPC1-silencing shRNA or control shRNA lentiviral construct. Cells were sorted, and knockdown was assessed by flow cytometry. Cells transduced with the shGPC1 construct showed a 47.2% reduction in GPC1 MFI relative to shScramble cells (Fig. 7F). Although the knockdown was incomplete, there was a decrease in MFI associated with a modest but significant reduction (37 ± 0.052%, *P* < 0.01) in HIV-1 viral yields in the shGPC1 compared to shScramble control cells (Fig. 7G).

### Glypican-1 overexpression is associated with increased HIV-1 infection of primary immune cells

**Fig 8:**
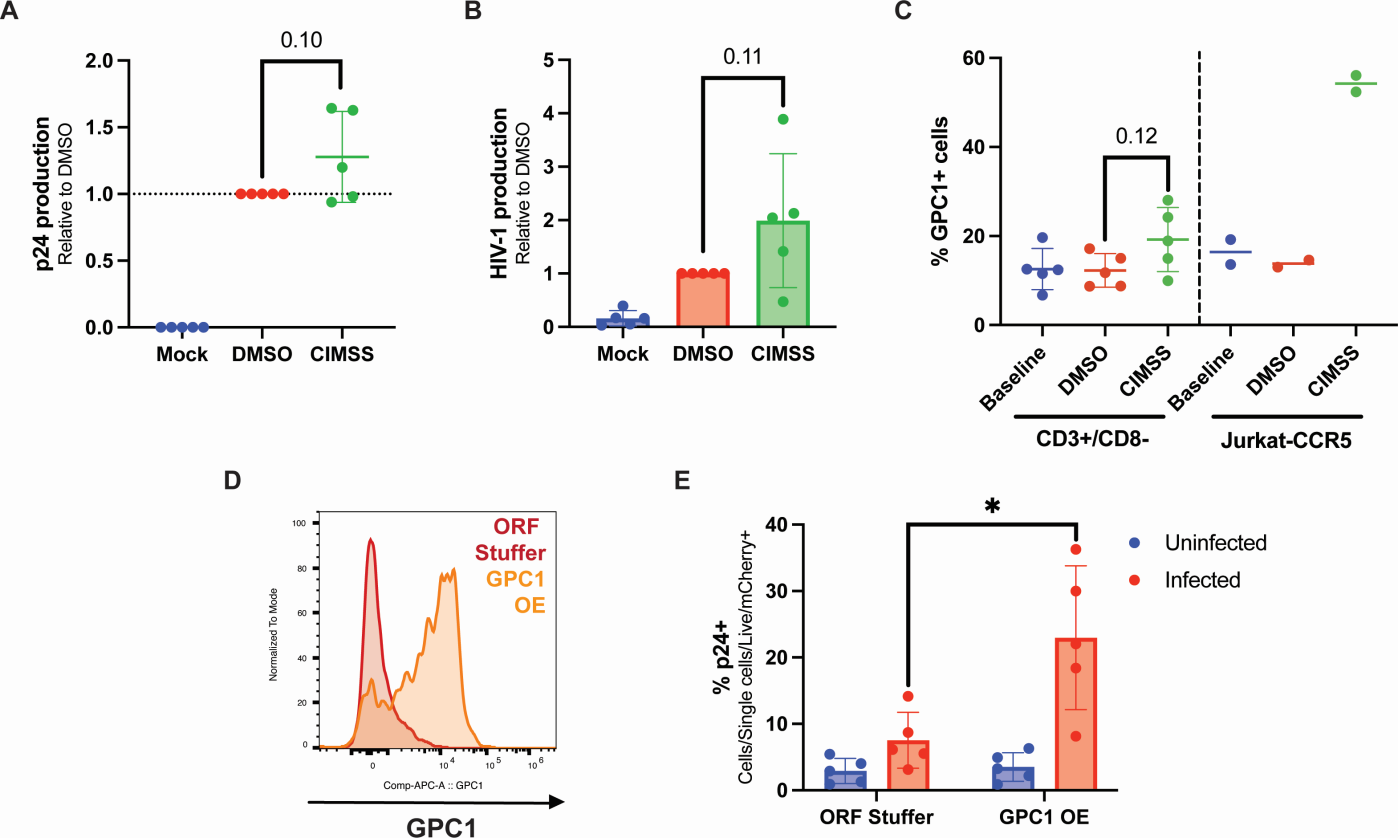
Alkyl-CIMSS treatment and glypican-1 overexpression increase HIV-1 infection of primary human CD4+ T cells. (**A**) PBMCs (*n* = 5) were treated with 50 μM alkyl-CIMSS or DMSO-vehicle control and infected for 5 days. At 5 dpi, viral production was quantified by measuring p24 in the cell supernatant by ELISA and (**B**) viral yields by TZM-bl luciferase assay in culture supernatants (*n* = 5). Results are presented as fold change relative to DMSO-treated cells. (**C**) The PBMCs were stained for CD3 and CD8 to identify CD4+ T cells and for GPC1 to quantify the percentage increase in GPC1 positive cells at baseline (uninfected cells) and in the HIV-infected cells treated with alkyl-CIMSS or DMSO vehicle control. The results are presented as the percentage of GPC1+ on the CD3^+^CD8^−^ cells (left). Similarly, Jurkat-CCR5 cells were infected with HIV in the presence of 50 μM alkyl-CIMSS or DMSO-vehicle control and stained for GPC1 after 3 days (*n* = 2, right). (**D**) Isolated primary CD4+ T cells were transduced with a full-length glypican-1 overexpression (GPC1 OE) or control (ORF stuffer) construct. GPC1 overexpression was assessed by flow cytometry on mCherry+ cells. (**E**) OE- or control-transduced cells were infected with HIV-1_BaL_ and assessed for infection by intracellular p24 measurement by flow cytometry (*n* = 5). Results are presented as p24+ cells of the mCherry+ population. All results are presented as mean ± SD and compared by unpaired *t*-test; *P < 0.05.

To assess whether the results obtained with Jurkat-CCR5 cells could be recapitulated in primary human cells, we treated PBMCs from five different donors with alkyl-CIMSS or control buffer prior to infecting with HIV-1_BaL_ (5 ng). A trend toward higher levels of p24 and viral yields (measured by TZM-bl assay of culture supernatants) was detected in alkyl-CIMSS compared to control-treated cells (Fig. 8A and B, *P* = 0.1). This increase was associated with enhanced GPC1 expression as monitored by flow cytometry in the alkyl-CIMSS but not DMSO-treated HIV-infected CD4+ cells (gating on CD3+/CD8^−^ subpopulation, *P* = 0.12) (Fig. 8C, left). Notably, the increase in GPC1 expression in response to alkyl-CIMSS in the primary cells was smaller than the response in Jurkat-CCR5 cells, which may contribute to the more modest increase in HIV infection (Fig. 8C, right).

To further address the effects of GPC1 on HIV-1 infection, we transduced primary CD4+ cells, isolated by negative selection from human PBMCs, with lentiviral constructs expressing either full-length GPC1 or a control ORF stuffer (Fig. 8D). The transduced cells were then infected with HIV-1_BaL_ (5 ng p24). The GPC1-transduced cells were significantly more susceptible to HIV infection (percentage of p24+ cells quantified by flow cytometry) than the control cells (*P* < 0.05, Fig. 8E), confirming a role for GPC1 in promoting HIV infection. However, we were unable to detect a difference in pSAMHD1 in the primary cells, which may reflect the heterogeneity of the cell population and cell cycle kinetics.

## DISCUSSION

Using TMEM16F KO cells, we confirmed that TMEM16F triggers PS translocation to the outer leaflet of the plasma membrane to promote HIV infection; however, the knockdown resulted in an incomplete loss of PS scrambling, suggesting possible contributions from other phospholipid translocases. However, unlike the cellular response to HSV or SARS-CoV-2, which activate PLSCR1, TMEM16F activation was not associated with Akt translocation. These results are consistent with a study of TMEM16F-dependent remodeling of the extracellularly exposed proteome in Jurkat cells, which also did not detect any increase in exofacial Akt in response to ionomycin (17). Given the differences in cellular responses to these two scramblases, we predicted that HIV would be insensitive to the inhibitory effects of alkyl-CIMSS. However, unexpectedly, alkyl-CIMSS increased HIV infection by both R5 and X4 viruses in TZM-bl cells and increased infection by R5 viruses in Jurkat-CCR5 cells.

Enhanced HIV infection was observed when the cells were pretreated with alkyl-CIMSS and washed prior to viral exposure, suggesting that the target(s) of alkyl-CIMSS is likely to be a molecule that is accessible at the cell surface, independent of virus-induced TMEM16F activation. The TMEM16F independence is supported by the observation that alkyl-CIMSS also increased HIV infection and upregulated GPC1 in the TMEM16F KO cells. These findings further highlight the differences in the target and cellular response to alkyl-CIMSS in the context of HIV compared to HSV infection. In the setting of HSV, inhibition is dependent on PLSCR1 activation and exofacial translocation of Akt and occurs during viral entry, whereas the enhancement of HIV infection by alkyl-CIMSS does not involve Akt translocation and occurs post-entry. There was no increase in bound or internalized HIV virus particles using a Vpr-EGFP reporter virus. In addition, increased HIV infection was observed when cells were infected with an HIV-1 virus pseudotyped with VSV-G, which binds and enters cells by a different pathway. Alkyl-CIMSS treatment, instead, increased early and late reverse transcription products.

**Fig 9:**
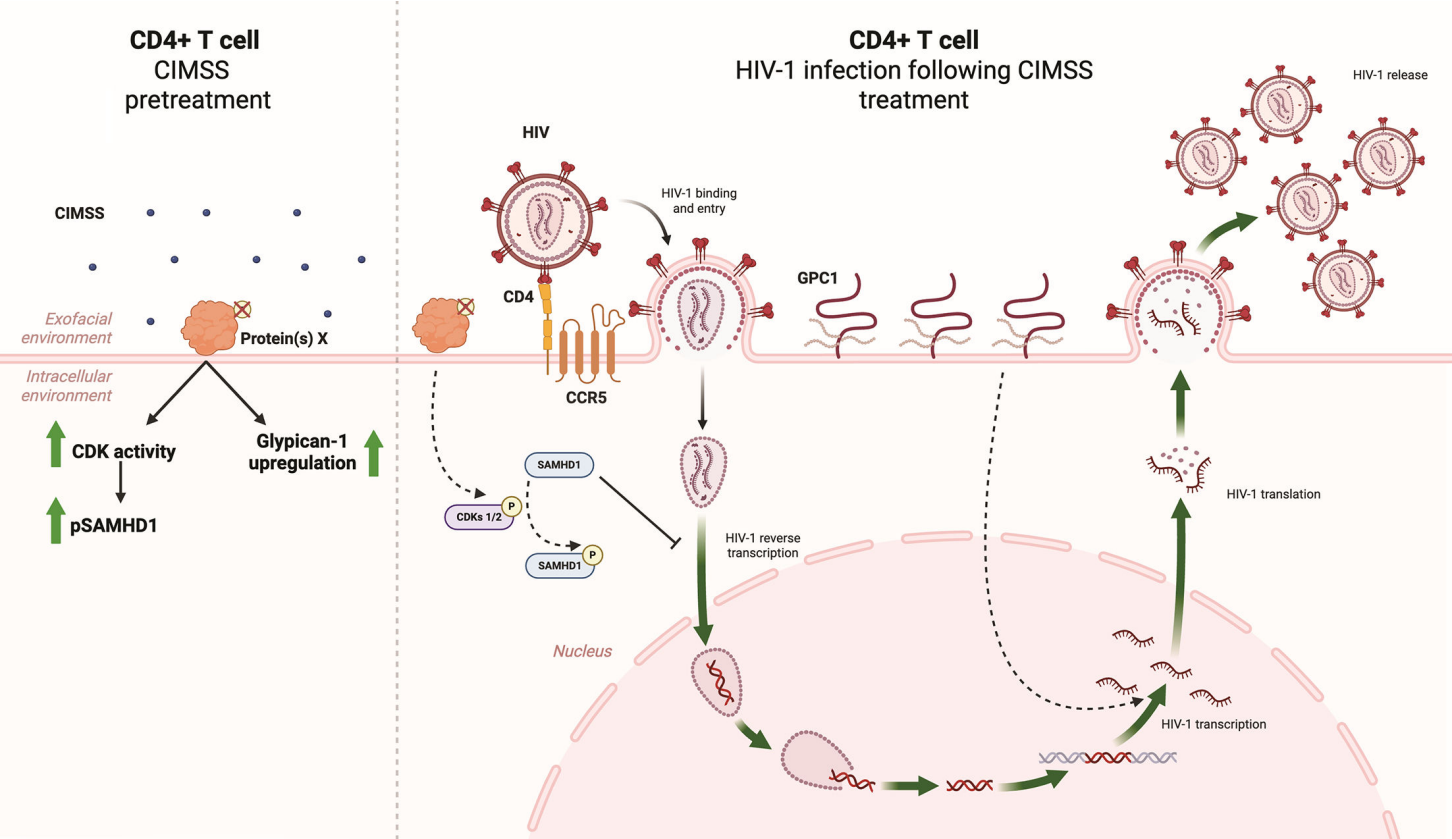
Model of Alkyl-CIMSS-mediated HIV-1 enhancement. Alkyl-CIMSS blocks the phosphorylation of exofacial protein(s), which promotes HIV-1 infection through two independent routes: increased CDK activity and the upregulation of glypican-1 (left panel). Increased CDK activity leads to higher levels of phosphorylated SAMHD1 at Thr592, resulting in increased reverse transcriptase (RT) products. The CDK inhibitor, flavopiridol, abrogates the enhancing effects of alkyl-CIMSS, reducing pSAMHD1 levels and RT products. The increase in GPC1 expression leads to increased HIV-1 transcription products and viral production.

Although we were unable to identify the target(s) of alkyl-CIMSS in Jurkat-CCR5 T cells, whole-cell phosphoproteomics detected an increase in CDK activity within 60 min of alkyl-CIMSS treatment. CDKs are intracellular proteins, suggesting that the increase in their activity may be part of an “outside-in” signaling response elicited by alkyl-CIMSS through its interactions with other molecules or kinases accessible on the outer leaflet of the plasma membrane (Fig. 9). Activation of CDKs in response to alkyl-CIMSS treatment was associated with phosphorylation of SAMHD1, and the addition of flavopiridol, a non-selective CDK inhibitor, blocked SAMHD1 phosphorylation and overcame the proviral effects of alkyl-CIMSS. This activity is consistent with our observation that alkyl-CIMSS increases early and late reverse transcription products, as phosphorylation of SAMHD1 releases its restriction of HIV reverse transcriptase, in part by modulating deoxyribonucleoside triphosphate levels (18, 19).

In addition to increased CDK activity, the whole-cell proteomic and transcriptomic studies identified increased expression of GPC1 in alkyl-CIMSS-treated cells. This result was confirmed by RT-qPCR, flow cytometry, and immunoblotting. Alkyl-CIMSS also upregulated GPC1 in TMEM16F KO cells, which partially restored their susceptibility to HIV-1 infection. The role of GPC1 in promoting HIV infection post-entry was confirmed by overexpressing the protein, which resulted in an increase in HIV transcription in response to both HIV-1_BaL_ and the VSV-G-pseudotyped virus. Importantly, overexpression of GPC1 in primary CD4+ T cells also significantly increased HIV infection. Alkyl-CIMSS treatment of PBMCs upregulated GPC1, which resulted in a trend toward increased HIV infection of CD4+ T cells. The magnitude of the increase in HIV infection was less than that observed in the Jurkat-CCR5 cell line, which may reflect heterogeneity of primary cells and a more modest increase in GPC1 following alkyl-CIMSS treatment.

Interestingly, the enhancing effects of alkyl-CIMSS and GPC1 overexpression on HIV infection were both inhibited by flavopiridol, suggesting that the upregulation of GPC1 may be linked to CDK activation. Consistent with this conjecture, GPC1 upregulation has been associated with higher levels of CDK activity in an *in vitro* cancer model (20). Since GPC1 was introduced using lentivirus constructs, we could not directly quantify reverse transcription products and instead relied on HIV LTR expression as an early marker of HIV infection. GPC1 functions as a co-receptor for a variety of signaling pathways, including TGF-β, VEGF, FGF, and Wnt (21–25), and may promote HIV-1 infection through multiple pathways, including HIV-1 transcription (26, 27).

Taken together, the results of these studies support the following model: alkyl-CIMSS targets molecule(s), presumably kinases, that are accessible on the outer leaflet of the plasma membrane, independent of TMEM16F activation. This leads to increased CDK activity and upregulation of GPC1 (Fig. 9). We propose that these responses act in concert to promote HIV infection by increasing reverse transcription products, possibly via overlapping pathways that are inhibited by flavopiridol.

The primary limitation of our study is that we were unable to identify the target(s) of alkyl-CIMSS driving these effects. Over 4,000 extracellularly exposed proteins were detected in resting Jurkat cells, and all but 465 were also detected in TMEM16F KO cells (17), including many canonically cytosolic kinases such as PKCα, CDK1, and CDK2, which may be targets of alkyl-CIMSS and mediate the “outside-in” signaling pathways modulated by alkyl-CIMSS treatment. We speculate that the target of alkyl-CIMSS driving HIV-1 infection is a kinase, given the presence of kinases on resting Jurkat cells and the potency of both alkyl-CIMSS and its parent molecule staurosporine as kinase inhibitors. Analogs of alkyl-CIMSS in development should facilitate the identification of its targets, allowing us to link intracellular changes with cell surface molecules. Identification of these “outside-in” pathways, which are activated in response to alkyl-CIMSS to promote HIV infection, may facilitate the future identification of novel targets for antiviral development.

## MATERIALS AND METHODS

### Cells

TZM-bl cells were obtained through the NIH HIV Reagent Program, Division of AIDS, NIAID, NIH. Jurkat-Tat-CCR5 (Jurkat-CCR5) cells were provided by Quentin Sattentau (Sir William Dunn School of Pathology, University of Oxford, Oxford, UK). PBMCs were obtained from HIV-uninfected donors (New York Blood Center), and CD4+ T cells were isolated using negative selection (EasySep Human CD4+ T cell Isolation Kit, 19,052, STEMCELL Technologies) and cultured in RPMI medium with 50 U/mL recombinant IL-2 (#202-IL, R&D Systems). Primary CD4+ T cells were activated for 72 h with 25 µL/mL ImmunoCult Human CD3/CD28 T cell activator (10971; STEMCELL Technologies). Whole PBMCs were cultured in complete RPMI medium.

TMEM16F knockout Jurkat CCR5 cells were generated using Dharmacon’s Edit-R CRISPR-Cas9 All-in-One lentiviral technology. Briefly, Jurkat-Tat-CCR5 cells were transduced with lentivirus containing Cas9, sgRNA targeting TMEM16F, and an EGFP selection marker at a multiplicity of infection (MOI) of 0.3 transducing units (TU) per cell. At 72 h post-transduction, single clones were isolated for outgrowth using the BD FACSAria. Gene expression and knockdown were validated by RT-qPCR and flow cytometry. Controls were generated by transducing cells with a non-targeting CRISPR/Cas9 control construct and following the same cloning strategy.

Jurkat-CCR5 cells overexpressing GPC1 were generated by lentiviral transduction using lentiviral vectors constructed by VectorBuilder. These pseudoviruses were produced by co-transfecting HEK293T cells in 10 cm^2^ dishes with 3 µg of plasmid DNA, 0.3 µg pMD2.G vector expressing VSV-G, and 2.5 µg pCMV∆8.2. FuGENE HD Transfection Reagent (Promega, E2311) was used at a 3:1 ratio to plasmid DNA. pCMVR8.74 (Addgene plasmid #22036) and pMD2.G (Addgene plasmid #12259) were gifts from Didier Trono. Pseudoviruses were added to Jurkat-CCR5 cells and incubated at 37°C for 3 days. After 3 days, mCherry-bright live cells were sorted using BD FACSAria for outgrowth. Gene expression and knockdown were validated by RT-qPCR and immunoblots. Control cells were stably transfected with an empty open reading frame and mCherry. A similar approach was used to generate Jurkat-CCR5 GPC1 knockdown cells by transducing the cells with lentiviruses expressing shRNA specific to GPC1 and mCherry or a shSramble shRNA and mCherry.

### Viruses and pseudoviruses

HIV-1_BaL_ (R5-utilizing strain) and HIV-1_iiiB_ (X4-utilizing strain) were acquired through the NIH AIDS Reagent Program, Division of AIDS, National Institute of Allergy and Infectious Diseases (NIAID), NIH, and grown on Jurkat (X4) or Jurkat-CCR5 (R5) cells for 12 days and stored at −80°C after filtration through a 0.2 μM filter. Viral stocks were quantified by p24 ELISA (R&D Systems, DY7360). Fluorescently labeled HIV-1 and pseudoviruses were produced by transfecting HEK293T in T150s using the ViaFect Transfection Reagent (Promega, E4981). EGFP labeled HIV-1 was produced by co-transfecting 60 µg pNL4-3_BaL_ p21-85 (NIH HIV Reagent Program, ARP-11441, contributed by Dr. Bruce Chesebro), 20 µg pEGFP-Vpr (NIH HIV Reagent Program, ARP-11386, contributed by Dr. Warner C. Greene), and 10 µg of pAdvantage (Promega, E1711). VSV-G-pseudotyped HIV-1 was produced by co-transfecting 60 µg of pNL4-3∆env (BEI Resources, NIAID, NIH, HRP-20281), 20 µg VSV-G expression vector (NIH HIV Reagent Program, ARP-4693, contributed by Dr. Lung-Ji Chang), and 10 µg pAdvantage. All HIV-based viruses were quantified by p24 ELISA (R&D Systems, DY7360). HSV-2 (G) and HSV-1(F-GS2822), which encodes red fluorescent protein fused to the N terminus of VP26 (28), were grown and titrated on Vero cells as previously described (29, 30).

### Synthesis of alkyl-CIMSS

Alkyl-CIMSS was synthesized in a similar manner to that used to synthesize CIMSS previously in our group (6, 31). Briefly, staurosporine was alkylated with methyl bromoacetate and saponified to afford known acid 2 (32–35). This acid was engaged in a HATU-mediated amide coupling with known sulfonate-containing amine S3 (6, 31) which, after neutralization, afforded alkyl-CIMSS S5 in its zwitterionic form. Full details of the synthesis of alkyl-CIMSS are found in Text S1.

### Flow cytometry to assess for phosphatidylserine and protein expression

Jurkat-CCR5 or TZM-bl cells were treated with either HIV-1_BaL_ (~10 ng p24/5 × 10^5^ cells) or HSV-2(G) (MOI 10) for 30 min or control buffer. The cells were then washed and stained with zombie NIR fixable viability dye (BioLegend, #423105). PS was detected using mouse anti-PS IgG, clone 1H6 (0.2 µg/mL, MilliporeSigma, #05-719) followed by goat-anti-mouse PE (1:200; Invitrogen, #31861). Akt was detected using anti-Akt (pan) from Cell Signaling (1:50, #5186). GPC1 was detected using anti-GPC1 (1:100, Invitrogen, PA5-28055) followed by goat-anti-rabbit APC (1:500, Invitrogen, A10931). HIV p24 and pSAMHD1 were detected in permeabilized cells with either anti-HIV1 p24 (1:250, NIH HIV Reagent Program, ARP-13449, contributed by DAIDS/NIAID) or anti-pSAMHD1(Thr592) (1:50, Cell Signaling, #87593S). Cells were fixed in 2% paraformaldehyde. Permeabilized cells generated using Cytofix/Cytoperm (BD, #554714) were used as staining controls. Samples were acquired on a Cytek Aurora flow cytometer (Cytek Biosciences) and analyzed with FlowJo (FlowJo LLC).

### Viral infection assays

Cells were infected with HSV-2(G) or HIV-1 (5 ng p24 unless otherwise indicated) for 2 h at 37°C. For non-adherent cells (Jurkat-CCR5 and PBMCs), the infected cells were washed and transferred to 24-well plates with 1 mL of complete media for 72 h. At 72 h, supernatants were harvested and stored at −80°C for TZM-bl assays, or the infected cells were permeabilized and assayed for p24 expression by flow cytometry. Alternatively, cells were lysed for RNA extraction and LTR expression. TZM-bl assays were conducted as previously described (36, 37). After 48 h of incubation, the cells were lysed in passive lysis buffer (Promega, #E1941), and luciferase activity was measured using the Promega assay system (Promega, #E1501).

PBMCs (2 × 10^6^ cells) were inoculated with 50 ng p24 in the presence of alkyl-CIMSS or DMSO. Every 2 days, half of the media was exchanged to resupplement vehicle or drug. At 5 days post-infection, media were collected and analyzed for p24 content by ELISA (R&D Systems, DY7360) or viral yield by TZM-bl assay. Values below the limit of detection were adjusted to 0.

Cells were infected with HSV-1(G) at MOI of 10 pfu/cell, and infection was monitored by flow cytometry for the immediate early gene ICP0 or RT-qPCR as described below.

### Pharmacologic inhibitors

TAK-779 (MedChemExpress, #HY-13406), AMD3100 (MilliporeSigma, 239825), CaCCinh-A01 (MedChemExpress, #HY-100611), Flavopiridol (MedChemExpress, #HY-10005), and DMSO (Cell Signaling, #12611) as a carrier control were added 5 min before application of virus. Concentrations used were determined non-toxic before use via MTS assay (Promega, G3582).

### RNA extraction and real-time quantitative reverse transcription-PCR (RT-qPCR)

Total RNA was extracted, cDNA was synthesized, and reverse-transcription polymerase chain reaction amplification was performed as described elsewhere (17). Primers/probes used were as follows: HSV ICP0 (forward: 5′-GGTCACGCCCACTATCAGGTA-3′; reverse: 5′-CCTGCACCCCTTCTGCAT-3′; probe: 5′-FAM-CAACGGAATCCAGGTCTTCATGCACG-TAMRA-3′); HIV LTR (forward: 5′-CACACAAGGCTACTTCCCTGA-3′; reverse: 5′-TCTCTGGCTCAACTGGTACTAGCTT-3′; probe: 5′-FAM-AGAACTACACACCAGGGCCAGGGATCAG-TAMRA-3′); TMEM16F (ANO6) (Thermo Fisher Scientific, Hs03805835_m1); GPC1 (Thermo Fisher Scientific, Hs00892476_m1); RPLP0 (Thermo Fisher Scientific, 4326314E), and GAPDH (Thermo Fisher Scientific, Hs02786624_g1). Targets were amplified in 10 μL reactions using TaqMan Gene Expression Master Mix (Applied Biosciences, #4369016) in a QuantStudio 7 Flex Real-Time PCR System (Thermo Fisher Scientific). Data were analyzed using QuantStudio software. Quantification was normalized against the housekeeping gene RPLP0 (T cell lines) or GAPDH (TZM-bl cells) in the same RNA extracts; relative gene expression was calculated using the 2^–ΔΔCt^ method.

### HIV-1 binding and entry assays

Cells were pretreated with 50 μM CIMSS or DMSO for 4 h at 37°C. Following this incubation, HIV_NL4.3BaL/VPR-EGFP_ (50 ng p24) was applied to the cells and allowed to incubate for 2 h at either 4°C (binding) or 37°C (entry). The cells were washed to remove any unbound virus and fixed for analysis by flow cytometry.

### Quantification of early and late HIV-1 reverse transcripts

Jurkat-CCR5 cells (10^5^) were pretreated with compounds for 15 min at 37°C before infection with 5 ng p24 HIV-1 BaL for 2 h. The cells were then washed thrice with media to remove any unbound virus and resuspended in complete media. At the indicated times, cells were washed with PBS and harvested for DNA extraction using the DNeasy Blood & Tissue Kit (QIAGEN, 69506). Twenty nanograms of genomic DNA was used per 10 μL real-time PCR reaction containing 2.5 nanomoles of probe and 5 nanomoles of each primer with TaqMan Gene Expression Master Mix (Applied Biosciences, #4369016). Primer/probes were as follows: Early Reverse Transcription product (ERT; forward: 5′- GTGCCCGTCTGTTGTGTGAC-3′; reverse: 5′- GGCGCCACTGCTAGAGATTT-3′; probe: 5′-FAM-CTAGAGATCCCTCAGACCCTTTTAGTCAGTGTGG-TAMRA-3′) and Late Reverse Transcription product (LRT; forward: 5′- TGTGTGCCCGTCTGTTGTGT-3′; reverse: 5′- GAGTCCTGCGTCGAGAGAGC-3′; probe: 5′-FAM- CAGTGGCGCCCGAACAGGGA-TAMRA-3′). Standard curves were generated by plotting Ct values against dilutions of pNL4-3 (NIH HIV Reagent Program, ARP-114, contributed by Dr. M. Martin) at known concentrations.

### Whole-cell proteomics

Jurkat-CCR5 cells were treated with 50 μM alkyl-CIMSS or 0.5% DMSO as a vehicle control before being either mock-infected or infected with HIV-1_BaL_ for 8 h. At 8 h, cell suspensions were transferred to conical tubes, pelleted, and resuspended in urea lysis buffer (8M urea, 50 mM NH4HCO3, 150 mM NaCl). For sample processing, Tris(2-carboxyethyl) phosphine (TCEP) was added to a final concentration of 4 mM. The DNA was sheared via probe sonication on ice at 20% amplitude for 20 s, followed by 10 s of rest, repeated three times. After sonication, protein concentration was determined using BCA assay (Thermo, 23225). Then, iodoacetamide (IAA) was added to each sample to a final concentration of 10 mM, and the samples were incubated in the dark at room temperature (RT) for 30 min. Excess IAA was quenched by the addition of dithiothreitol (DTT) to 10 mM, followed by incubation in the dark at RT for 30 min. Samples were then diluted with 0.1 M NH4HCO3 (pH = 8.0) to a final urea concentration of 2 M. Trypsin (Gold mass spectrometry grade, Promega) was added at a 1:100 (enzyme:protein, wt:wt) ratio and digested overnight at 37 °C with rotation. Following digestion, 10% trifluoroacetic acid (TFA) was added to each sample to a final pH ~2. Samples were desalted under vacuum using Sep Pak tC18 cartridges (Waters). Each cartridge was activated with 1 mL 80% acetonitrile (ACN)/0.1% TFA and equilibrated with 3 × 1 mL of 0.1% TFA. Following sample loading, cartridges were washed with 4 × 1 mL of 0.1% TFA, and samples were eluted with 4 × 0.5 mL 50% ACN/0.25% formic acid (FA). Eluted peptides were vacuum centrifuged to dryness.

To prepare phosphoenrichment beads, 30 µL per sample of 50% Ni-NTA Superflow bead slurry (QIAGEN) was added to a 2 mL empty spin column (Bio-Spin, Bio-Rad). Beads were washed three times with 1 mL of HPLC-grade water, incubated four times with 1 mL of 100 mM EDTA for 30 s, washed three times with 1 mL of HPLC-grade water, incubated four times with 1 mL of 15 mM FeCl_3_ for 1 min, washed three times with 1 mL of HPLC-grade water, and washed once with 1 mL of 0.5% (vol/vol) FA. Beads were resuspended in 750 µL of 80% ACN/0.1% TFA. One milligram of digested peptides was resuspended in 83.33 µL of 40% ACN/0.1% TFA and 166.67 µL of 100% ACN/0.1% TFA, and 60 µL of the bead slurry was added to each sample and incubated for 30 min while rotating at RT. A C18 BioSPN column (Nest Group), centrifuged at 110 × *g* for 1 min for each step, was equilibrated two times with 200 μL of 80% ACN/0.1% TFA. Beads were loaded onto the column and washed four times with 200 μL of 80% ACN/0.1% TFA, then washed three times with 200 μL of 0.5% FA. Then, 200 μL of 500 mM potassium phosphate buffer pH 7 was added three times to the column and incubated for 1 min. Then, 200 μL of 0.5% FA was added three times to the column. Phosphopeptides were eluted twice with 100 μL of 40% ACN/0.1% FA and vacuum-centrifuged to dryness. Phosphopeptides were resuspended in 25 µL of 4% FA/3% ACN for mass spectrometry analysis.

All samples were analyzed on an Orbitrap Eclipse mass spectrometry system equipped with an Easy nLC 1200 ultra-high-pressure liquid chromatography system interfaced via a Nanospray Flex nanoelectrospray source (Thermo Fisher Scientific). Samples were injected onto a fritted fused silica capillary (30 cm × 75 µm inner diameter with a 15 μm tip, CoAnn Technologies) packed with ReprosilPur C18-AQ 1.9 μm particles (Dr. Maisch GmbH). Buffer A consisted of 0.1% formic acid in water, and buffer B consisted of 0.1% formic acid in 80% acetonitrile. Peptides were separated using an organic gradient from 5% to 35% mobile buffer B over 120 min, followed by an increase to 100% B over 10 min at a flow rate of 300 nL/min. Analytical columns were equilibrated with 3 μL of buffer A.

Data were acquired in a data-independent analysis (DIA) manner. A full scan was collected at 60,000 resolving power over a scan range of 390–1,010 m/z, with an instrument-controlled AGC target, an RF lens setting of 30%, and an instrument-controlled maximum injection time, followed by DIA scans using 8 m/z isolation windows over 400–1,000 m/z at a normalized HCD collision energy of 28%.

For proteome analysis, the DIA-NN algorithm was used to identify peptides/proteins and extract intensity information from DIA data (38). A library-free FASTA digest was used for library generation using the *Homo sapiens* UniProt reference proteome (downloaded on August 23, 2023) and HIV-1 (strain NL4-3) protein sequences. Search settings were the algorithm defaults: the protease setting was Trypsin/P, maximum missed cleavages was 1, fixed modifications for N-terminal methionine excision and cysteine carbamidomethylation were included, the peptide length was 7–30 amino acids, the precursor charge range was 1–4, the precursor m/z range was 300–1,800, the fragment ion m/z range was 200–1,800, and the precursor false discovery rates were set to 1%.

For phosphoproteome analysis, the Spectronaut algorithm (version 15.2) was used to build spectral libraries from DDA data, identify peptides/proteins, localize phosphorylation sites, and extract intensity information from DIA data (39). All data were searched against the *Homo sapien*s UniProt reference proteome (downloaded on August 23, 2023) and HIV-1 (strain NL4-3) protein sequences. All data were filtered to achieve a false discovery rate of 0.01 for peptide-spectrum matches, peptide identifications, and protein identifications. Search parameters included a fixed modification for carbamidomethyl cysteine and variable modifications for N-terminal protein acetylation, methionine oxidation, and serine, threonine, and tyrosine phosphorylation. Peptide lengths from 7 to 52 amino acids were considered, and up to two missed cleavages were allowed. MS2 b- and y-ion types were utilized.

Statistical analysis of proteomics data was conducted utilizing the MSstats package in R (40). All data were normalized by equalizing median intensities; the summary method was Tukey’s median polish; the maximum quantile for deciding censored missing values was 0.999, and only the top 25 most abundant features per protein were included in modeling.

### Kinase activity analysis

Kinase activity analysis was performed with the KSEA package in R, using log_2_ fold-change values to rank phosphorylation sites and the ProtMapper database of kinase-substrate interactions (41, 42). Only kinase-substrate interactions with a belief score of 1 were used for this analysis. Phosphorylation sites identified by peptides that were not unique to a single protein sequence and phosphorylation sites detected on multiply phosphorylated peptides were excluded from this analysis.

### Bulk RNA sequencing

Jurkat-CCR5 cells were treated with 50 μM alkyl-CIMSS or 0.5% DMSO as a vehicle control and then infected with HIV-1_BaL_ or control media for 8 h. The cells were then lysed, and RNA was extracted using the RNeasy Plus Mini kit (QIAGEN, #74134). Isolated RNA was sent to Azenta Life Sciences (New Jersey, USA) for library preparation and sequencing on the Illumina NovaSeq platform using 150 bp paired-end reads to generate 30 million read pairs per sample. Raw sequence data were processed using the nf-core RNA-Seq pipeline (version 3.18.0) (43, 44). Reads were aligned to the GRCh38 human reference genome using the STAR aligner, and gene quantification was performed against the GENCODE v42 primary assembly annotation with RSEM to obtain gene expression counts (45, 46). Expressed genes were determined using zFPKM (47). Normalization and differential expression analysis were performed using DESeq2 (48). Gene set enrichment analysis was performed using clusterProfiler and GSVA (49, 50).

### Western blots

Total cell lysates were prepared from equal cell numbers using the radioimmunoprecipitation assay (RIPA) buffer system (Santa Cruz, sc-24948) containing 1× phosphatase and protease inhibitor cocktail (Thermo Fisher, 78440). SDS-PAGE gel electrophoresis was performed on a gradient polyacrylamide gel (Bio-Rad, 5671083) for 80 min at 120 V and transferred on PVDF (Bio-Rad, 1704157) using the Turboblot (Bio-Rad, 1704150). Western blots were visualized and scanned using the ChemiDoc imaging system (Bio-Rad). Protein loading was compared by staining with an antibody for GAPDH (1:1,000, Cell Signaling, 2118).

### Statistical analyses and data sharing

Statistical analyses of RT-qPCR data were performed using log10-transformed values, including where data are presented as non-transformed values. Data were tested for normality and analyzed using parametric (normally distributed) or non-parametric (non-normally distributed) tests. *P*-value < 0.05 was considered significant. Analyses were performed using GraphPad Prism version 10.5.0 software (GraphPad Software Inc., San Diego, CA).

## Data Availability

The RNA-seq data sets generated in this study have been deposited in the NCBI Gene Expression Omnibus (GEO) under accession number GSE325970. The input data and scripts used to generate the figures and numbers reported in this article were deposited to the GitHub repository: https://github.com/cutleraging/2025-Kelsey-Vinzant.

## EDITOR'S NOTE

**Ed. Note**: A potential conflict of interest was identified post-acceptance, and the editor in chief conducted an additional review to ensure the integrity and fairness of the peer review process.

## SUPPLEMENTAL MATERIAL

Text S1 jvi.00160-26-s0001.docx Full details of the synthesis of Alkyl-CIMSS.

Supplemental material jvi.00160-26-s0002.docx Figures S1 to S4 and Table S1.

Table S2 jvi.00160-26-s0003.xlsx Phosphoproteomic dataset from cells treated with Alkyl-CIMSS for 30 and 60 min.

Table S3 jvi.00160-26-s0004.xlsx Transcriptomic and proteomic datasets from cells treated with Alkyl-CIMSS in the absence of HIV-1.

Table S4 jvi.00160-26-s0005.xlsx Transcriptomic and proteomic datasets from cells treated with Alkyl-CIMSS in the presence of HIV-1.

ASM does not own the copyrights to Supplemental Material that may be linked to, or accessed through, an article. The authors have granted ASM a non-exclusive, world-wide license to publish the Supplemental Material files. Please contact the corresponding author directly for reuse.
