# Supplementary material for: A cell-impermeable kinase inhibitor uncovers outside-in signaling pathways that promote HIV-1 infection
Source: J Virol. 2026 May 11;100(6):e00160-26. doi: 10.1128/jvi.00160-26 (PMC13288470; doi:10.1128/jvi.00160-26)
Supplement: Text S1 — Full details of the synthesis of Alkyl-CIMSS. [file jvi.00160-26-s0001.docx]

**Supplemental Text 1: Full details of the synthesis of alkyl-CIMSS.**

**A. Description of the synthesis of Alkyl-CIMSS**

Alkyl-CIMSS was synthesised in a similar manner to that used to synthesise CIMSS previously in our group.^1, 2^ Briefly, staurosporine was alkylated with methyl bromoacetate and saponified to afford known acid **2^3-6^**. This acid was engaged in a HATU-mediated amide coupling with known sulfonate-containing amine **S3**^1, 2^ which, after neutralisation, afforded alkyl-CIMSS **S5** in its zwitterionic form.

**B. Schematic of Alkyl-CIMSS synthesis**

**C. General experimental details**

Staurosporine and its analogues are light sensitive and prolonged light exposure caused degradation, which can be observed by a “yellowing” of material and the appearance of extra peaks in the ^1^H and ^13^C NMR spectra. Accordingly, all procedures were carried out in the absence of light and analysis was undertaken using amber NMR tubes and LCMS vials. Reactions requiring anhydrous conditions were carried out in oven-dried glassware under a positive pressure of argon in anhydrous solvents using standard Schlenk techniques. Anhydrous solvents were purchased from Sigma Aldrich and used as received. Reaction progress was monitored by thin layer chromatography (TLC) on Merck Aluminum-backed silica gel coated TLC plates (60 Å, F_254_ indicator). TLC plates were visualized by exposure to ultraviolet light (254 nm) and KMnO_4_ or Cerium Molybdate (Hanessian's Stain). Flash column chromatography was performed with a Büchi Pure C815 Flash automated flash chromatography system using prepacked FlashPure cartridges containing either silica gel (50 μm irregular) or C18 silica gel (50 μm spherical), using ACS grade solvents. All yields refer to chromatographically and spectroscopically (^1^H and ^13^C{1H} NMR) pure material unless otherwise stated. NMR spectra were recorded using a Bruker 500 MHz spectrometer. All chemical shifts (δ) are reported in parts per million (ppm) and referenced to residual protium or the carbon resonance of the NMR solvent, respectively. Data are represented as follows: chemical shift, multiplicity (br = broad, s = singlet, d = doublet, t = triplet, q = quartet, m = multiplet), coupling constants (*J*) in Hertz (Hz), integration. High resolution electrospray ionization (ESI) mass spectra were undertaken on a Waters QTOF Premier Tandem Mass spectrometer fitted with a Waters 2795 HPLC. The purity of Alkyl-CIMSS **S5** was assessed by LCMS performed on an Agilent 1260 Infinity II Series LC System with an Agilent 6120B Single Quadrupole LC/MS (ESI/QTOF), equipped with an Agilent 1100 Multi Wavelength Detector and an Agilent Infinity II 1290 Evaporative Light Scattering Detector with a linear gradient system. Column, solvents, and flow rate are given below the relevant spectrum.

**D. Experimental Procedures:**

**Methyl *N*-[(5*R,*7*R,*8*R,*9*S*)-8-methoxy-9-methyl-16-oxo-6,7,8,9,15,16-hexahydro-5*H*,14*H*-17-oxa-4b,9a,15-triaza-5,9-methanodibenzo[*b,h*]cyclonona[*jkl*]cyclopenta[*e*]-*as*-indacen-7-yl]-*N*-methylglycinate (S1):**

Staurosporine (250 mg, 536 µmol) was dissolved in dry DMF (18 mL) under an atmosphere of argon. Potassium carbonate (370 mg, 2.68 mmol) and methyl bromoacetate (0.50 mL, 5.4 mmol) were added sequentially and the resulting suspension was stirred for 16 hours at room temperature after which time LCMS analysis indicated complete consumption of starting material. The suspension was poured into water (30 mL), resulting in a white precipitate. This was cooled to 0 °C for 15 minutes, then filtered. The fine, sticky precipitate was washed once with water (5 mL) before being dissolved in acetonitrile (10 mL) and concentrated under reduced pressure. The material was then co-evaporated with acetonitrile (10 mL) twice more to remove any residual water, before being dried under high vacuum to yield **S1** (258 mg, 89%) as a white foam,^3, 5-7^ which was used directly in the next step without further purification.

^1^H NMR (500 MHz, CDCl_3_) δ 9.44 (d, *J* = 7.9 Hz, 1H), 7.92 (d, *J* = 7.2 Hz, 1H), 7.87 (d, *J* = 8.5 Hz, 1H), 7.49 (ddd, *J* = 8.2, 7.1, 1.2 Hz, 1H), 7.44 (ddd, *J* = 8.5, 7.1, 1.3 Hz, 1H), 7.38 (ddd, *J* = 8.1, 7.1, 1.0 Hz, 1H), 7.34 (ddd, *J* = 7.9, 7.1, 0.8 Hz, 1H), 7.29 (d, *J* = 8.2 Hz, 1H), 6.63 (dd, *J* = 7.0, 2.6 Hz, 1H), 6.21 (s, 1H), 5.04 (s, 1H), 4.00 (d, *J* = 2.3 Hz, 1H), 3.74 – 3.67 (m, 1H), 3.52 (s, 3H), 3.06 (d, *J* = 17.7 Hz, 1H), 3.00 (s, 3H), 2.72 (ddd, *J* = 14.2, 7.8, 2.7 Hz, 1H), 2.64 – 2.55 (m, 2H), 2.40 (s, 3H), 1.93 (s, 3H) (1 H not observed). HRMS (ESI, +ve) *m/z*: [M + H]^+^ calcd for C_31_H_31_N­_4_O_5_ 539.2294, found 539.2297. These data are consistent with those reported in the literature.^5^

***N*-[(5*R,*7*R,*8*R,*9*S*)-8-Methoxy-9-methyl-16-oxo-6,7,8,9,15,16-hexahydro-5*H*,14*H*-17-oxa-4b,9a,15-triaza-5,9-methanodibenzo[*b,h*]cyclonona[jkl]cyclopenta[*e*]-*as*-indacen-7-yl]-*N*-methylglycine (S2):**

Ester **S1** (258 mg, 479 µmol) was dissolved in MeOH (16 mL) and sodium hydroxide (4.8 mL of a 1 M aqueous solution, 4.8 mmol) was added. The solution was heated at 45 °C for 2.5 h, after which time LCMS analysis indicated complete consumption of starting material. The solution was cooled to room temperature and carefully neutralised to pH 7 with 1 M HCl. The solvents were removed under reduced pressure, and the residue was adsorbed onto celite, before being purified by automated column chromatography (silica gel, 0 🡪 30% water/MeCN v/v) to afford **S2** (176 mg, 70%) as a white solid.^3, 4, 6^

^1^H NMR (500 MHz, DMSO-d_6_) δ 9.29 (d, *J* = 7.7 Hz, 1H), 8.54 (s, 1H), 8.04 (s, 1H), 8.02 (s, 1H), 7.59 (d, *J* = 8.2 Hz, 1H), 7.47 (ddd, *J* = 8.3, 7.0, 1.3 Hz, 2H), 7.33 (dd, *J* = 8.0, 6.9 Hz, 1H), 7.28 (ddd, *J* = 8.0, 7.0, 1.0 Hz, 1H), 6.81 (dd, *J* = 7.5, 3.0 Hz, 1H), 4.99 (d, 1H), 4.98 (d, 1H), 4.25 (d, *J* = 2.2 Hz, 1H), 3.61 (ddd, *J* = 9.0, 4.6, 2.1 Hz, 1H), 2.97 (d, *J* = 17.6 Hz, 1H), 2.86 (s, 3H), 2.76 (ddt, *J* = 12.0, 7.5, 4.6 Hz, 1H), 2.64 (d, *J* = 17.6 Hz, 1H), 2.43 – 2.38 (m, 1H), 2.36 (s, 3H), 1.93 (s, 3H) (proton for carboxylic acid not observed); ^13^C NMR (126 MHz, DMSO-d_6_) δ 172.5, 172.1, 138.7, 136.3, 132.2, 129.9, 126.3, 125.6, 125.1, 124.8, 123.8, 122.5, 121.3, 120.0, 119.2, 119.1, 114.4, 113.7(5), 113.7(2), 108.7, 93.4, 83.7, 81.3, 58.8, 54.4, 53.6, 45.4, 39.5 (obscured by solvent peak), 29.0, 28.4; HRMS (ESI, –ve) *m/z*: [M – H]^–^ calcd for C_30_H_27_N­_4_O_5_ 523.1981, found 523.1976.

**3-{4-[(2-{(*R*)-[(5*R,*7*R,*8*R,*9*S*)-8-Methoxy-9-methyl-16-oxo-6,7,8,9,15,16-hexahydro-5*H*,14*H*-17-oxa-4b,9a,15-triaza-5,9-methanodibenzo[*b,h*]cyclonona[*jkl*]cyclopenta[*e*]-*as*-indacen-7-yl](methyl)ammonio}acetamido)methyl]-1*H*-1,2,3-triazol-1-yl}propane-1-sulfonate (S5):**

Dry DMF (7.5 mL) was added to a flask containing carboxylic acid **S2** (98 mg, 190 µmol), amine **S3^1, 2^** (52 mg, 220 µmol) and HATU (85 mg, 220 µmol), and the suspension was stirred for 5 minutes at room temperature. DIPEA (39 µL, 220 µmol) was added in one portion and the solids dissolved to form a clear, light-yellow solution, which was stirred at room temperature for 42 h (Note: generally, this reaction was complete after 12 h). Upon reaction completion, the solvent was removed under reduced pressure at 25 °C. Water (10 mL) was added, followed by sodium carbonate (234 µL of a 1 M aqueous solution, 234 µmol) to form a clear solution. This was concentrated under reduced pressure at 25 °C, followed by further drying under high vacuum for 30 minutes. The resulting sodium salt **S4** was purified by automated reversed-phase column chromatography (C18 silica gel, 5 🡪 80% MeCN/water v/v). Concentration of the product-containing fractions (eluted at 30% MeCN/water v/v) afforded 110 mg of sodium salt **S4**. This solid was redissolved in water (3 mL) with the aid of sonication, and HCl (1.47 mL of a 0.1 M aqueous solution, 0.147 mmol, 1 equiv.) was added dropwise, immediately forming a fine white precipitate. The precipitate was filtered and washed with cold water (2 × 3 mL). The resulting solid was suspended in water and lyophilised to afford **S5** (102 mg, 75%) as a white solid.

^1^H NMR (500 MHz, CD_3_OD, *ca.* 1 mg Na_2_CO_3_ (*ca.* 7 equiv) added) δ 9.28 (d, *J* = 8.0 Hz, 1H), 8.05 (d, *J* = 8.5 Hz, 1H), 7.96 (d, *J* = 7.8 Hz, 1H), 7.55 – 7.45 (m, 3H), 7.39 – 7.29 (m, 2H), 7.27 (s, 1H), 6.71 (dd, *J* = 7.1, 2.4 Hz, 1H), 4.98 (d, *J* = 2.7 Hz, 2H), 4.47 (hept, *J* = 6.9 Hz, 2H), 4.22 (d, *J* = 2.4 Hz, 1H), 4.08 (d, *J* = 15.5 Hz, 1H), 3.87 (d, *J* = 15.5 Hz, 1H), 3.38 – 3.34 (m, 2H), 3.03 (s, 3H), 2.87 – 2.76 (m, 2H), 2.75 – 2.68 (m, 3H), 2.38 (s, 3H), 2.27 (dt, *J* = 13.8, 6.8 Hz, 2H), 1.84 (s, 3H) (NH protons not observed); ^13^C NMR (126 MHz, CD_3_OD, *ca* 1 mg Na_2_CO_3_ added) δ 175.2, 173.5, 145.9, 140.7, 138.4, 133.9, 131.6, 127.9, 127.3, 126.8, 126.3, 125.6, 124.3, 123.4, 122.2, 121.8, 120.9, 120.0, 116.6, 115.6, 115.5, 109.3, 94.7, 86.3, 82.9, 59.7, 59.1, 57.0, 50.0, 47.2, 42.2, 35.0, 30.8, 29.3, 27.1 (1 carbon not observed); HRMS (ESI, +ve*) m/z*: [M + H]^+^ calcd for C_36_H_39_­N_8_O_7_S 727.2662, found 727.2663.

**E. NMR Spectra**

^1^H NMR spectrum (500 MHz, CDCl_3_) of methyl ester **S1**

^1^H NMR spectrum (500 MHz, DMSO-d_6_) of carboxylic acid **S2**

^13^C NMR spectrum (126 MHz, DMSO-d_6_) of carboxylic acid **S2**

^1^H NMR spectrum (500 MHz, CD_3_OD) of Alkyl-CIMSS **S5**

^13^C NMR spectrum (126 MHz, CD_3_OD) of Alkyl-CIMSS **S5**

**LCMS spectrum** of compound Alkyl-CIMSS **S5**.


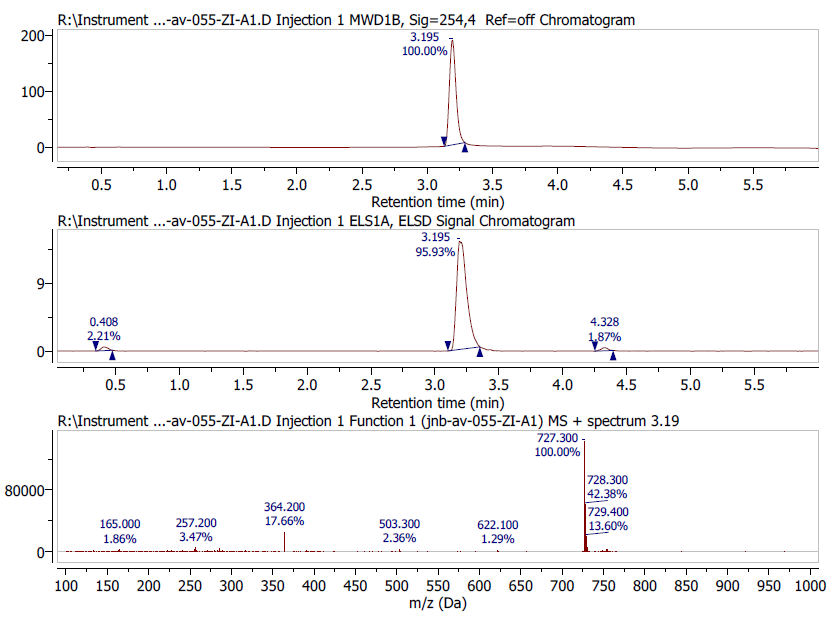


Column: C18 Kinetex 3 × 50 mm 2.6 micron. Flow rate: 1 mL/min. Method: 30% MeOH/water (+0.1% formic acid) for 0.5 min, then 30 – 100% MeOH/water (+0.1% formic acid) over 3.0 minutes followed by 100% MeOH for 1.5 minutes. Run finishes back at 30% MeOH/water (+0.1% formic acid). Purity assessed by ELSD and UV absorption at 254 nm.

**References**

1. Cheshenko, N.; Bonanno, J. B.; Hoffmann, H. H.; Jangra, R. K.; Chandran, K.; Rice, C. M.; Almo, S. C.; Herold, B. C., Cell-impermeable staurosporine analog targets extracellular kinases to inhibit HSV and SARS-CoV-2. *Commun Biol* **2022,** *5* (1), 1096.

2. Herold, B. C.; Almo, S. C. Pharmaceutical composition for treating severe acute respiratory syndrome coronavirus 2 and/or herpes simplex virus. WO2023086547, 2023.

3. Caravatti, G.; Fredenhagen, A. Preparation of N-acyl- and N-hydrocarbylstaurosporines as protein kinase C inhibitors. EP296110, 1988.

4. Caravatti, G.; Meyer, T.; Fredenhagen, A.; Trinks, U.; Mett, H.; Fabbro, D., Inhibitory activity and selectivity of staurosporine derivatives towards protein kinase C. *Bioorganic & Medicinal Chemistry Letters* **1994,** *4* (3), 399-404.

5. Li, Z.; Qian, L.; Li, L.; Bernhammer, J. C.; Huynh, H. V.; Lee, J. S.; Yao, S. Q., Tetrazole Photoclick Chemistry: Reinvestigating Its Suitability as a Bioorthogonal Reaction and Potential Applications. *Angew Chem Int Ed Engl* **2016,** *55* (6), 2002-6.

6. Rouhani, R.; Vainshtein, I. Screening for enzyme inhibitors using indicator enzyme fragment-inhibitor conjugates and active indicator enzyme-forming second enzyme fragment. WO2002090593, 2002.

7. Disney, A. J.; Kellam, B.; Dekker, L. V., Alkylation of Staurosporine to Derive a Kinase Probe for Fluorescence Applications. *ChemMedChem* **2016,** *11* (9), 972-9.
