# Supplementary material for: A cell-impermeable kinase inhibitor uncovers outside-in signaling pathways that promote HIV-1 infection
Source: J Virol. 2026 May 11;100(6):e00160-26. doi: 10.1128/jvi.00160-26 (PMC13288470; doi:10.1128/jvi.00160-26)
Supplement: Supplemental material — Figures S1 to S4 and Table S1. [file jvi.00160-26-s0002.docx]

**Supplemental Material**

**
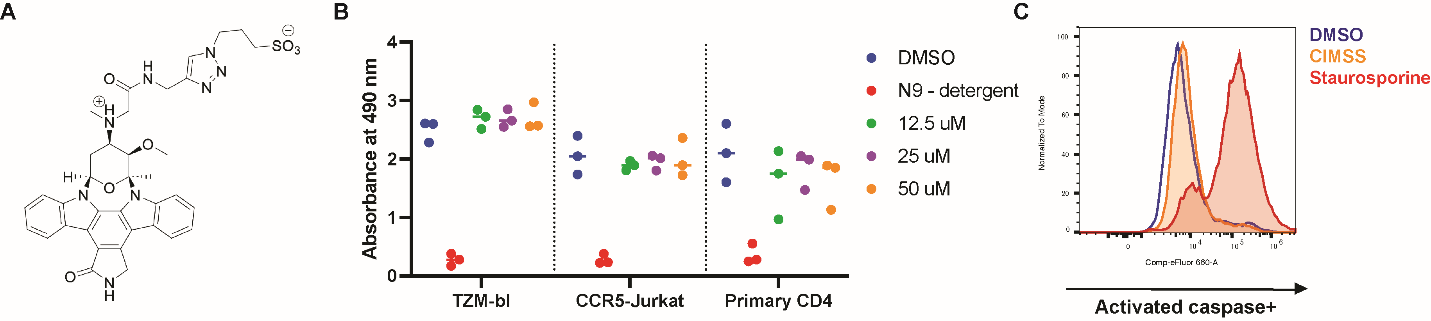
**

**S1 Fig. Alkyl-CIMSS is non-toxic across cell types and does not trigger caspase activation.**

(A) Model of alkyl-CIMSS. (B) CCR5-Jurkat cells (~50% confluence) were cultured in media containing increasing concentrations of Alkyl-CIMSS (12.5–50 μM) or the equivalent highest concentration of DMSO (0.5%) and cell proliferation and viability quantified after 24 h (n=3 independent experiments conducted in triplicate). (C) CCR5-Jurkat cells treated with either DMSO (blue), 50 μM Alkyl-CIMSS (orange), or 10 μM staurosporine (red) for 6 hours. Representative histogram (N=2) depicts poly-caspase activation as measured by flow cytometry.

**
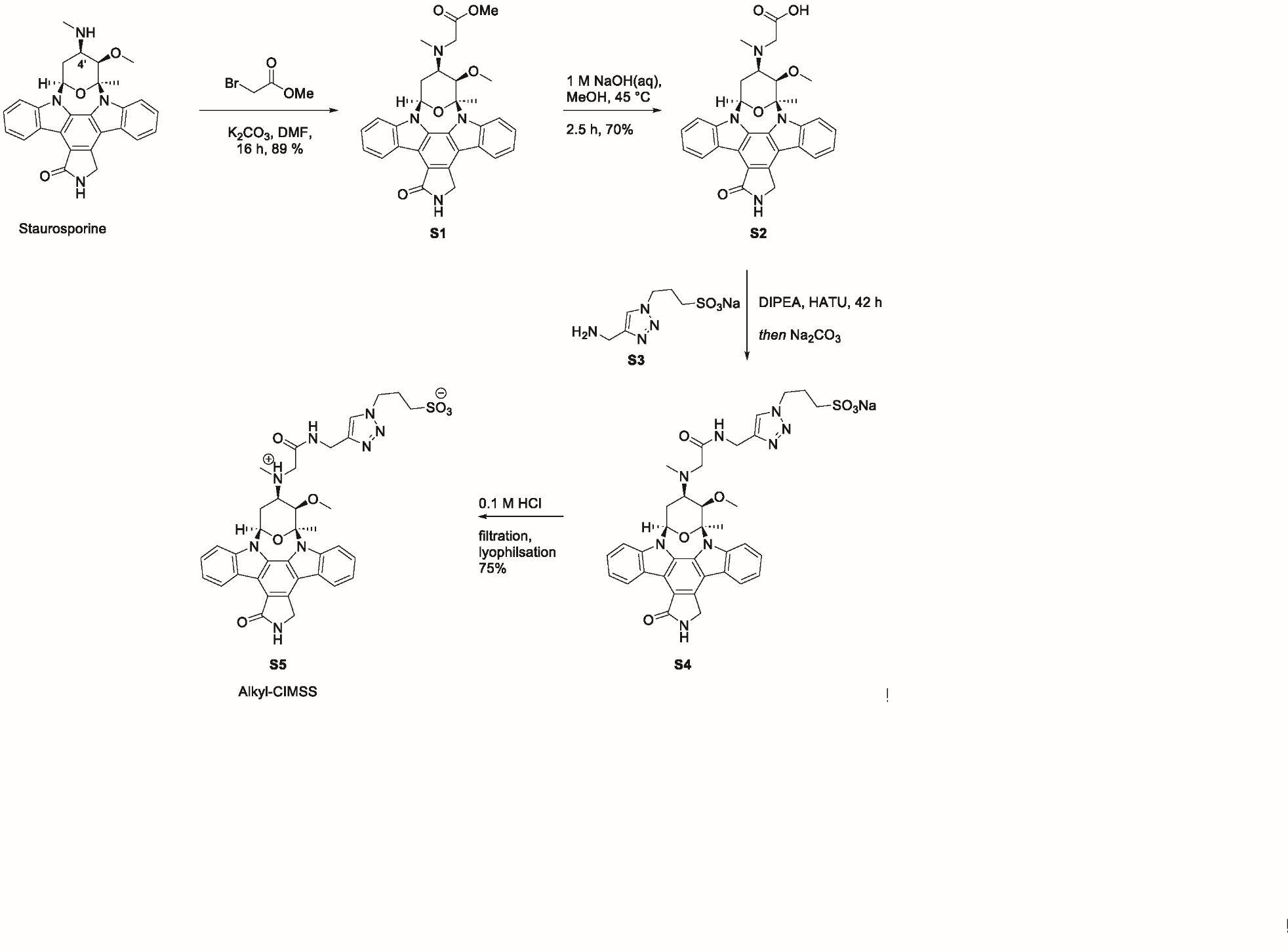
**

**S2 Fig. Synthesis of Alkyl-CIMSS.**

Staurosporine was alkylated with methyl bromoacetate and saponified to afford known acid **2**. This acid was engaged in a HATU-mediated amide coupling with known sulfonate-containing amine **S3** which, after neutralization, afforded alkyl-CIMSS **S5** in its zwitterionic form.

**
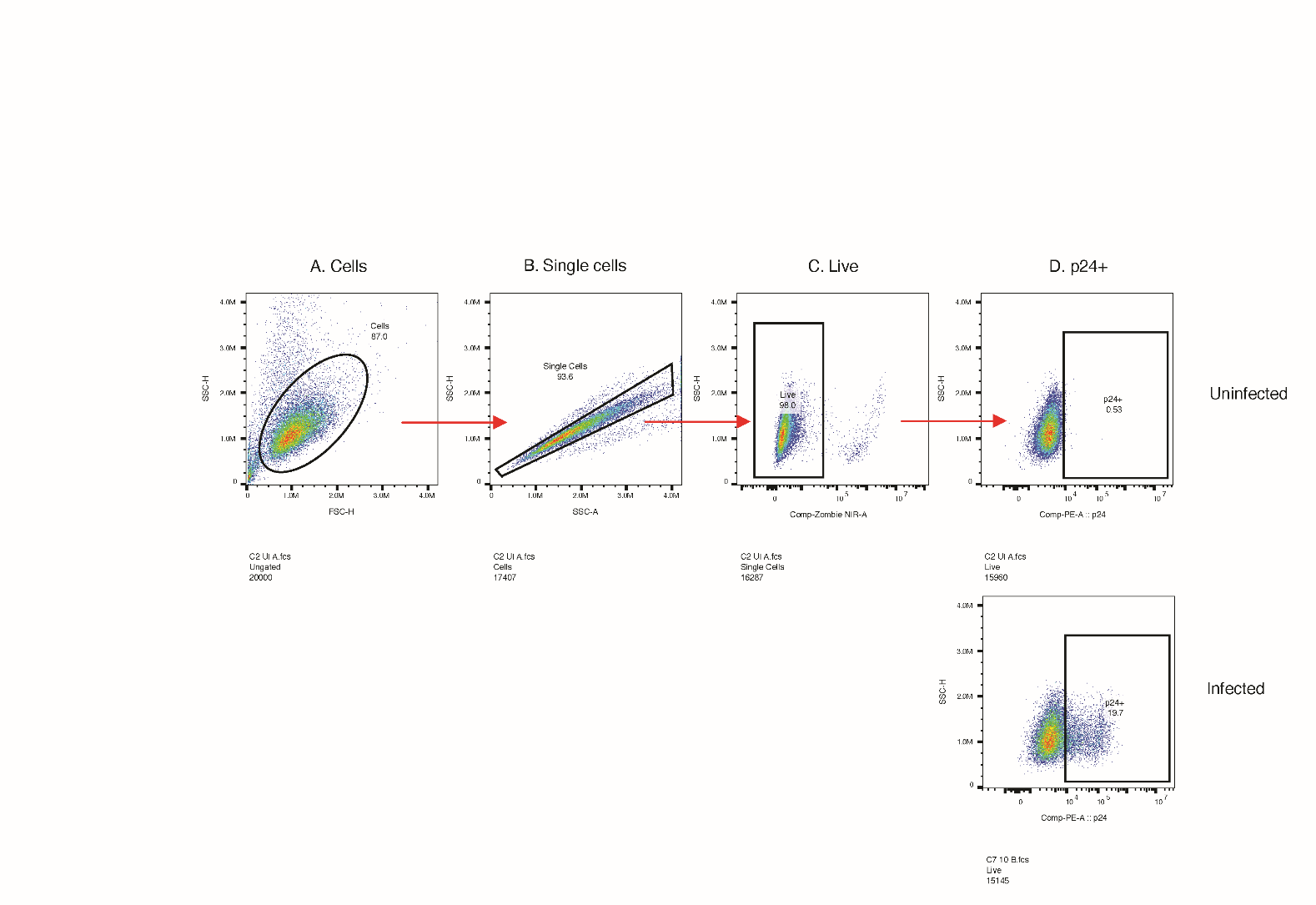
**

**S3 Fig. Gating strategy used for the flow cytometry analysis of HIV-1_BaL_ infection of CCR5-Jurkat cells.**

(A) Single cells were selected by gating SSC-A vs SSC-H (B). (C) Live vs dead cells were determined with the Zombie NIR fixable viability kit. (D) Cells were further analyzed for intracellular p24 expression.

**
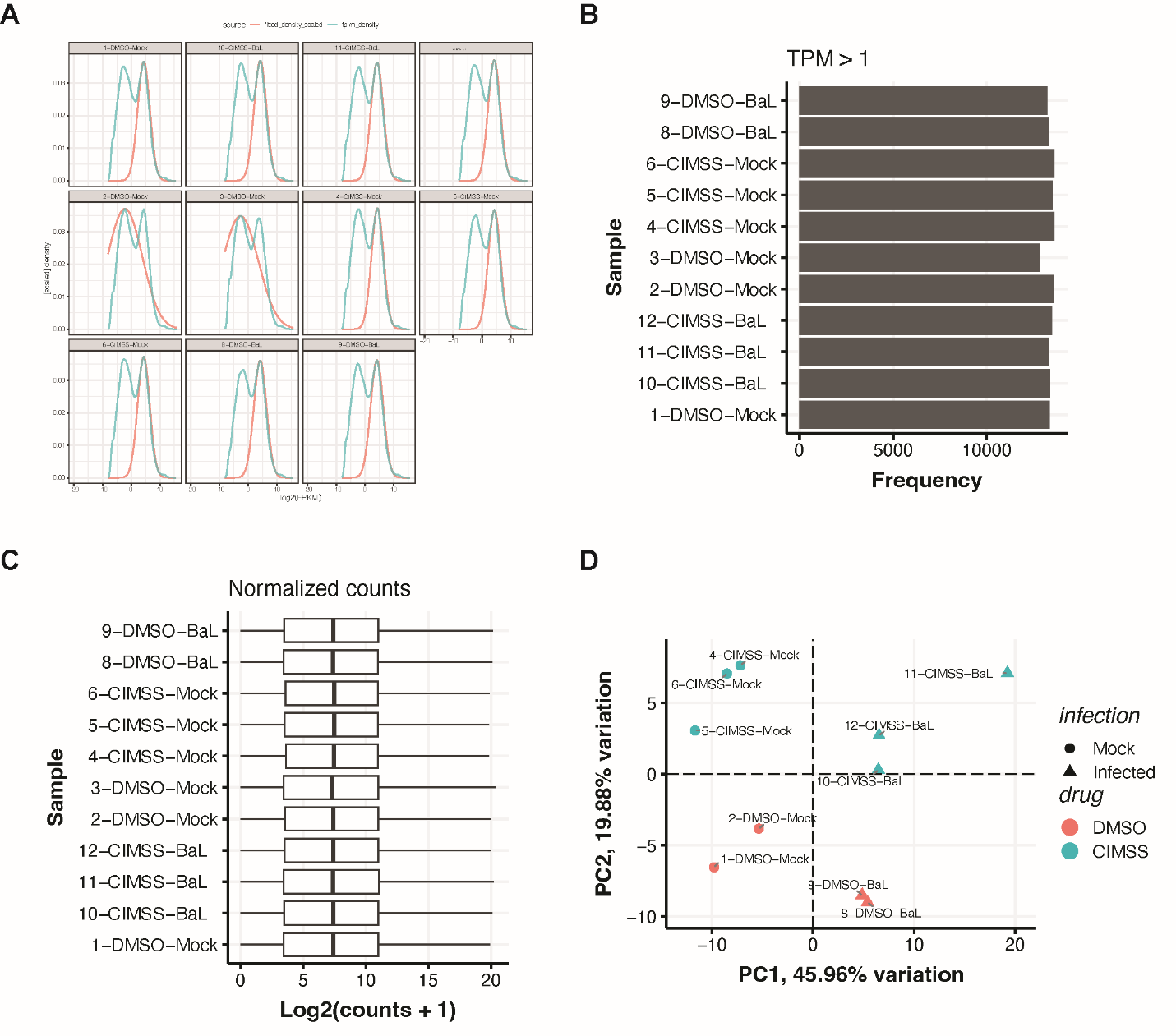
**

**S4 Fig. Transcriptomics quality control.**

(A) Density plots Log_2_(FPKM) of each sample showing unfiltered distribution (blue) and the Gaussian fit to the Log_2_(FPKM) data, which represents the filtering threshold used to identify expressed genes used in downstream analyses. (B) Number of genes detected at TPM > 1 in each sample. (C) Distribution of DESeq2 normalized Log2(counts + 1). (D) Principle component analysis (PCA) of all samples showing first 2 PCs.

**S1 Table. Biochemical characterization of Alkyl-CIMSS.**

(A) Permeability of Alkyl-CIMSS as determined by MDCK-MDR1 assay. (B) Enzyme activity in the presence of Alkyl-CIMSS.

**S2 Table. Phospho-proteomic dataset from cells treated with Alkyl-CIMSS for 30 and 60 minutes.**

**S3 Table. Transcriptomic and proteomic datasets from cells treated with Alkyl-CIMSS in the absence of HIV-1.**

**S4 Table. Transcriptomic and proteomic datasets from cells treated with Alkyl-CIMSS in the presence of HIV-1.**

**S1 Text: Full details of the synthesis of Alkyl-CIMSS.** (A) Description and (B) schematic of Alkyl-CIMSS synthesis. (C) General experimental details and (D) experimental procedures for Alkyl-CIMSS synthesis. (E) NMR spectra.
